# Glycosaminoglycans and Sialylated Glycans Sequentially Facilitate Merkel Cell Polyomavirus Infectious Entry

**DOI:** 10.1371/journal.ppat.1002161

**Published:** 2011-07-28

**Authors:** Rachel M. Schowalter, Diana V. Pastrana, Christopher B. Buck

**Affiliations:** Tumor Virus Molecular Biology Section, Laboratory of Cellular Oncology, National Cancer Institute, Bethesda, Maryland, United States of America; University of Michigan, United States of America

## Abstract

Merkel cell polyomavirus (MCV or MCPyV) appears to be a causal factor in the development of Merkel cell carcinoma, a rare but highly lethal form of skin cancer. Although recent reports indicate that MCV virions are commonly shed from apparently healthy human skin, the precise cellular tropism of the virus in healthy subjects remains unclear. To begin to explore this question, we set out to identify the cellular receptors or co-receptors required for the infectious entry of MCV. Although several previously studied polyomavirus species have been shown to bind to cell surface sialic acid residues associated with glycolipids or glycoproteins, we found that sialylated glycans are not required for initial attachment of MCV virions to cultured human cell lines. Instead, glycosaminoglycans (GAGs), such as heparan sulfate (HS) and chondroitin sulfate (CS), serve as initial attachment receptors during the MCV infectious entry process. Using cell lines deficient in GAG biosynthesis, we found that N-sulfated and/or 6-O-sulfated forms of HS mediate infectious entry of MCV reporter vectors, while CS appears to be dispensable. Intriguingly, although cell lines deficient in sialylated glycans readily bind MCV capsids, the cells are highly resistant to MCV reporter vector-mediated gene transduction. This suggests that sialylated glycans play a post-attachment role in the infectious entry process. Results observed using MCV reporter vectors were confirmed using a novel system for infectious propagation of native MCV virions. Taken together, the findings suggest a model in which MCV infectious entry occurs via initial cell binding mediated primarily by HS, followed by secondary interactions with a sialylated entry co-factor. The study should facilitate the development of inhibitors of MCV infection and help shed light on the infectious entry pathways and cellular tropism of the virus.

## Author Summary

Strong evidence suggests that Merkel cell polyomavirus (MCV or MCPyV) is a causative factor in the development of a large proportion of cancers arising from epidermal Merkel cells. While Merkel cell carcinoma is rare, it appears that infection with MCV is common, and many healthy people chronically shed MCV virions from the surface of their skin. In an effort to better understand the factors controlling MCV tissue tropism, we sought to characterize the cellular receptors that mediate MCV attachment to cultured cells. Several previously-examined polyomaviruses utilize sialic acid-containing glycolipids and glycoproteins to mediate cell binding and infectious entry. Our results show that, in contrast to other polyomaviruses, MCV does not require sialic acid-bearing glycans for attachment to cells, but instead uses a different group of carbohydrates called glycosaminoglycans for the initial attachment step of the infectious entry process. Interestingly, although sialic acid-bearing glycans are dispensable for initial attachment to cells, data using cells deficient in sialylated glycans suggest that sialic acids may form an essential element of a possible co-receptor that is engaged after the initial attachment of MCV to the cell via glycosaminoglycans.

## Introduction

The viral family *Polyomaviridae* consists of a diverse group of non-enveloped DNA viruses that infect humans as well as a range of other vertebrates. The family name is derived from the observation that murine polyomavirus causes tumors in various tissues in experimentally infected animals. The apparently broad tissue tropism of murine polyomavirus is consistent with the widespread distribution of its primary infectious entry receptors, a group of sialic acid-bearing glycolipids known as gangliosides [1]. Other well-studied polyomaviruses, such as the human polyomavirus BKV and its close relative, simian virus-40 (SV40), also employ gangliosides for infectious entry into cells (reviewed in [2]). Another BKV relative, JCV, has recently been shown to bind a specific sialylated pentasaccharide, known as LSTc, that decorates either proteins or gangliosides on a restricted range of cell types [3]. This is consistent with the much narrower cellular tropism of JCV [4], [5].

Although it has been suggested that initial attachment to sialic acid residues may be a universal infectious entry step for all polyomaviruses, the infectious entry pathways used by most members of the family have not yet been extensively investigated. Members of other non-enveloped virus families, such as the *Parvoviridae*, have been found to use a wide range of cellular receptors. For example, the primary cellular attachment receptors for adeno-associated viruses (AAVs) of the parvovirus genus *Dependovirus* range from gangliosides (bovine AAV; [6]), to protein-linked sialic acids (AAV4 and 5; [7]) or a very different type of carbohydrate side-chain, heparan sulfate (AAV2; [8]) (reviewed in [9]). Heparan sulfate (HS) is a type of glycosaminoglycan (GAG) that rarely contains sialic acid [10], but is instead characterized by specific patterns of N- and O-linked sulfate modifications [11]. AAV6 can bind to both sialylated polysaccharides and to HS on cells, and both interactions appear to modulate transduction into various tissues [12], [13]. In light of the *Dependovirus* precedent, the hypothesis that all polyomaviruses use sialic acid residues for initial attachment to cells should be viewed with caution.

Seven of the nine polyomavirus species known to infect humans were discovered within the past four years [14], [15], [16], [17], [18], [19]. Perhaps the most intriguing of these new discoveries is a human polyomavirus species named Merkel cell polyomavirus (MCV or MCPyV). MCV is believed to play a causal role in Merkel cell carcinoma (MCC), a highly lethal form of skin cancer (reviewed in [20]). An emerging view is that, unlike BKV and JCV, which commonly infect the urinary epithelium, MCV establishes a chronic productive infection in the skin of most adults [17], [21], [22]. It remains unclear which of the dozen or so different cell types that can be found in the skin are the primary source of shed MCV virions.

In an initial effort to better understand the cellular tropism of MCV, we set out to determine which receptors mediate initial attachment of the virion to the cell surface. A previous report by Erickson, Garcea and Tsai showed that recombinant MCV VP1 capsid protein subunits produced in bacteria can bind sialylated components of cell extracts, including the ganglioside GT1b [23]. Erickson and colleagues' data support speculation that MCV might follow an entry pathway similar to that of BKV, which has been shown to require GT1b or related complex gangliosides for infectious entry [24]. To further investigate this hypothesis, we employed MCV- and BKV-based reporter vectors (also known as pseudoviruses) as models for infectious entry into cultured cell lines [25]. To confirm the reporter vector-based results, we developed a system for titering the infectivity of native MCV virions.

Our results support a model in which MCV uses GAGs, likely in the form of HS, as initial attachment receptors. The initial GAG-mediated binding appears to be followed by interactions between the MCV virion and sialylated host cell factors. The use of GAGs, such as HS, as attachment receptors for MCV infectious entry is strikingly reminiscent of a different family of non-enveloped viruses, the *Papillomaviridae*, which are exclusively tropic for keratinocytes, a cell type that forms the epidermal layers of the skin and mucosa. The results suggest a possible example of convergent adaptation to exploitation of the epidermis as an infectious niche.

## Results

### Hemagglutination activities of MCV and BKV capsids

**Figure 1:**
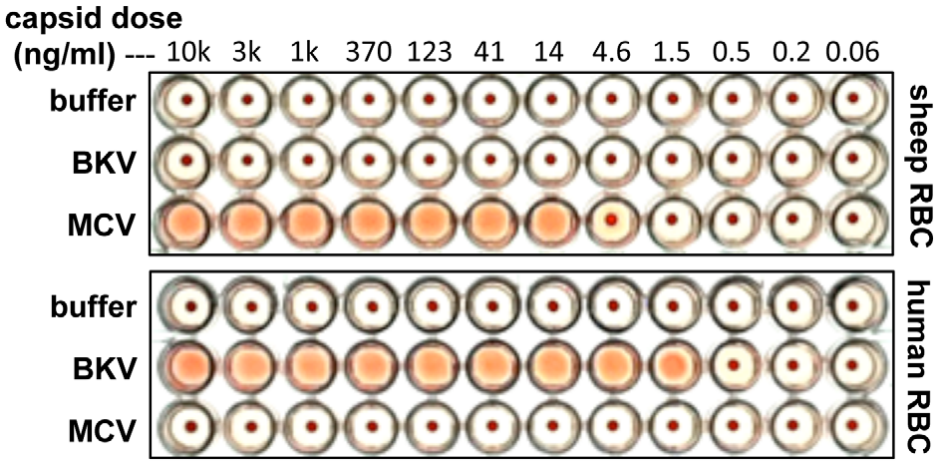
Hemagglutination assays. Serial dilutions of BKV or MCV capsids were mixed with sheep or human red blood cells (RBCs) in PBS and allowed to settle in round-bottom wells at 4°C overnight.

Hemagglutination assays (HA) are a classic method for investigating the interaction of virions with the cell surface. Hemagglutination is typically mediated by interactions between virion surface proteins and sialylated glycans displayed on red blood cells (RBCs). Erickson and colleagues have previously shown that recombinant MCV VP1 capsid protein subunits produced in bacteria can hemagglutinate sheep RBCs [23]. Using purified, fully-assembled MCV capsids produced in human cells (see below) [25], [26] we confirmed Erickson and colleagues' sheep RBC HA results (Figure 1). In contrast to MCV, BKV capsids did not display HA activity against sheep RBCs.

Many virus types show differential abilities to hemagglutinate RBCs from different animal species. This is thought to reflect differences in the display of different forms of sialylated glycans or other binding targets on the surface of RBCs from different animals [27]. BKV HA assays typically employ human RBCs [28]. Consistent with previous results, recombinant BKV capsids induced robust HA of human RBCs. In contrast, MCV capsids showed a surprising lack of HA activity against human RBCs (Figure 1). The results show that MCV and BKV engage mutually distinct attachment factors on RBCs.

### Sialylated glycans are dispensable for MCV attachment to cultured cells but required for a post-attachment entry step

When the MCV major and minor capsid proteins (VP1 and VP2, respectively) are co-expressed in human embryonic kidney-derived 293TT cells, they can spontaneously co-assemble around transfected reporter plasmids [26]. This results in the formation of reporter vector particles (also known as pseudovirions) that physically resemble native polyomavirus virions [26] and are capable of delivering encapsidated reporter plasmids to fresh target cells [25]. Similar systems have been developed for production of reporter vectors based on other polyomaviruses [5], [29] and for papillomaviruses [30]. Using MCV, BKV and human papillomavirus type 16 (HPV16) reporter vectors, we sought to identify candidate receptor or co-receptor molecules that are used by MCV for infectious entry into cultured cells.

In a comparative analysis of MCV and BKV reporter vector transduction efficiency in over sixty different cell lines from various human tumors, we determined that the human lung epithelial cell line A549 was among the most MCV-transducible lines in the panel (unpublished results). A549 cells were chosen for initial experiments because they also have the convenient feature of being readily transducible with BKV and HPV16 reporter vectors, allowing comparisons to these better-studied virus types.

**Figure 2:**
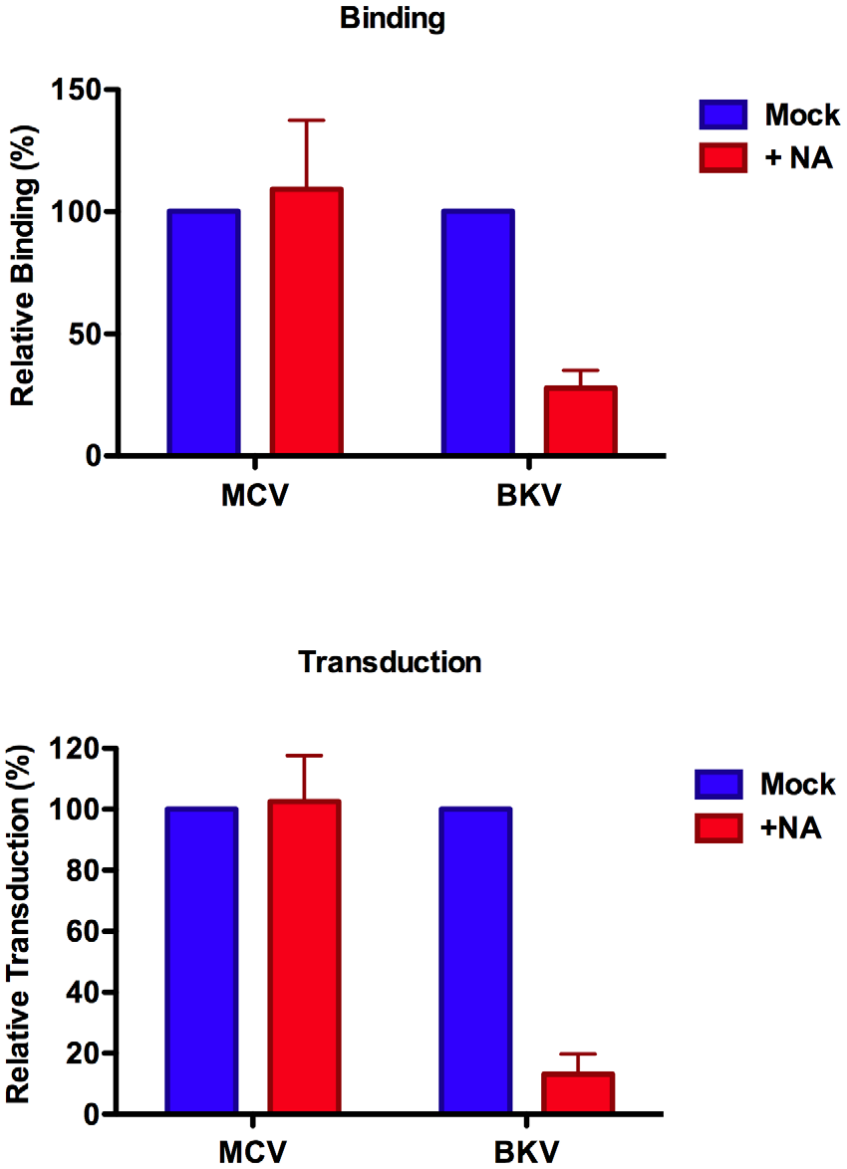
Neuraminidase treatment of A549 cells. The binding of Alexa Fluor 488-conjugated capsids (top panel) or reporter vector-mediated delivery of a GFP reporter gene (bottom panel) to A549 cells treated with neuraminidase was measured by flow cytometry. Results were standardized to mock-treated A549 cells. The average of three separate experiments is shown and error bars represent the standard deviation. See also Figure S2, which shows unstandaradized raw data for an individual experimental replicate.

To examine the binding of MCV or BKV capsids to cultured cells, we conjugated recombinant capsids to Alexa Fluor 488 to allow monitoring of cell binding by flow cytometry. The fluorochrome-conjugated capsids exhibited HA titers similar to unconjugated capsids (Figure S1A), suggesting that the dye conjugation process did not cause dramatic alterations in the cell binding properties of the capsids. Similarly, the dye conjugation procedure did not significantly affect the transducing potential of MCV reporter vectors (Figure S1B). In an initial series of experiments, we examined the binding of fluorochrome-conjugated MCV and BKV capsids to A549 cells. As shown in Figure 2 (and Figure S2), the binding of MCV to A549 cells was not significantly affected by pre-treatment of the cells with a broad-spectrum neuraminidase from *Arthrobacter ureafaciens* that is capable of hydrolyzing most forms of sialic acid linkage [31]. BKV capsid binding to A549 cells was, as expected, sensitive to neuraminidase. The transduction of a GFP reporter plasmid into A549 cells via MCV or BKV vectors in the presence or absence of neuraminidase mirrored the binding results (Figure 2 and Figure S2).

Keratinocytes and melanocytes are the two most abundant cell types in the epidermal layer of the skin. Based on the speculative assumption that MCV might productively infect one of these cell types in vivo, we examined a variety of melanocyte and keratinocyte-derived cell lines for transducibility with MCV, BKV and HPV16 reporter vectors. The human melanoma-derived line SK-MEL-2, as well as primary adult human epidermal keratinocytes (HEKa cells) were found to be readily transducible with both MCV and BKV reporter vectors. Neuraminidase treatment of both SK-MEL-2 cells and HEKa cells resulted in inhibition of BKV transduction, but had little effect on MCV transduction (Figure S3), consistent with results observed using A549 cells.

**Figure 3:**
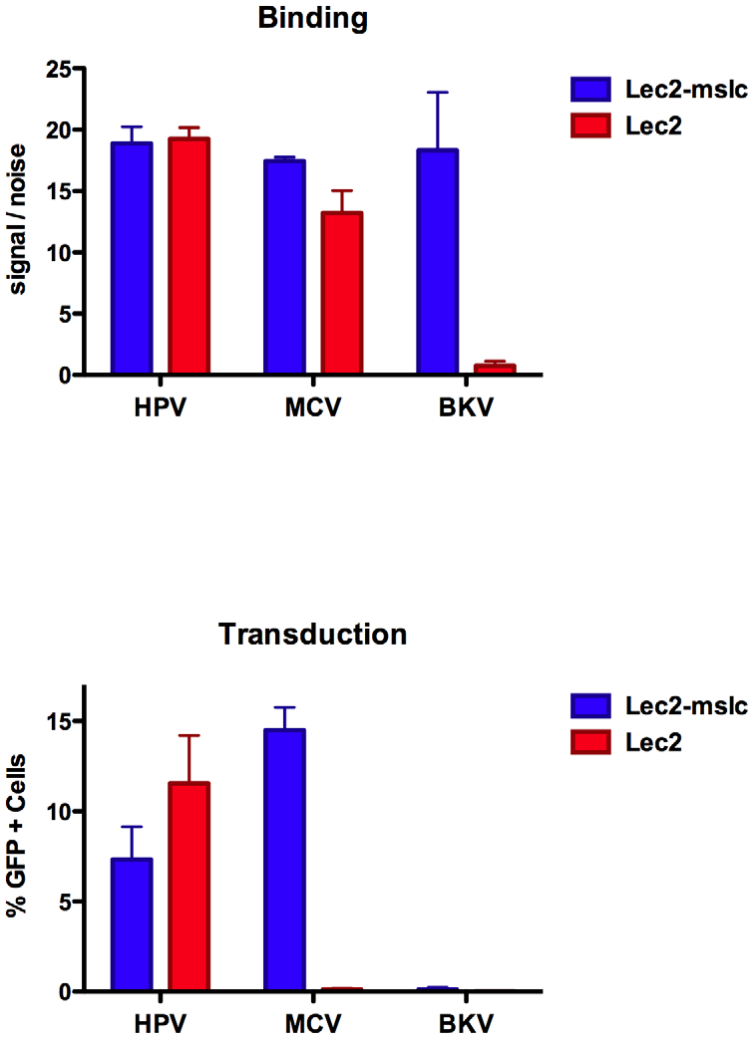
Binding and transduction of sialic acid-deficient cells. Binding of Alexa Fluor 488-labeled capsids (top panel) or transducing activity of reporter vectors (bottom panel) on sialic acid-deficient Lec2 cells or Lec2 cells stably expressing the sialic acid transporter SLC35A1 (Lec2-mslc). The average values and standard error of the mean from three separate experiments are shown.

It is known that some sialic acids, such as the single sialic acid residue on the ganglioside GM1 (which serves as a receptor for SV40), are resistant to digestion with neuraminidase [32]. To address the possibility that MCV attachment to cells is mediated by a sialylated glycan that is resistant to neuraminidase, we used a cell line deficient in biosynthesis of sialylated glycans. The line, known as Lec2, is a Chinese hamster ovary (CHO)-based mutant that lacks a functional gene for SLC35A1, a CMP-sialic acid transporter required for sialylation of glycoprotein and glycolipid ectodomains in the lumen of the Golgi [33]. A control line, Lec2-mslc, was engineered to stably express a wild-type SLC35A1 allele. As seen in Figure 3, restoration of the SLC35A1 gene resulted in a 12-fold increase in BKV capsid binding, confirming that the introduced gene restored the production of sialylated glycans. In contrast to BKV, there was no effect on HPV16 and only a slight improvement in MCV binding to the Lec2-mslc line. The results indicate that MCV capsid attachment to this cell line is largely independent of sialylated glycans.

All CHO-based cell lines are deficient in complex gangliosides, such as GT1b [34], [35]. Thus, restoration of the SLC35A1 sialic acid carrier to Lec2 cells would only be expected to restore sialylation of proteoglycans and simple gangliosides. Consistent with their lack of complex gangliosides, parental CHO-K1 cells (data not shown) and the CHO-based Lec2 cells with or without the restored SLC35A1 gene are highly resistant to BKV transduction (Figure 3). Despite the fact that MCV capsids readily bind to Lec2 cells, the line was surprisingly resistant to transduction by MCV reporter vectors (Figure 3). Reintroduction of the functional SLC35A1 allele rendered the line permissive for MCV transduction. A simple model that could explain the results would be that, while sialylated factors are not required for the initial attachment of MCV to the cell surface, the virus appears to require sialylation of a cellular factor for an entry step that occurs after stable attachment to the cell. The ability of MCV to transduce CHO-K1 and Lec2-mslc cells suggests that complex gangliosides are not necessary for MCV transduction. Indeed, while pre-treatment of cells with exogenous GT1b rescued BKV transduction of Lec2 cells, exogenous GT1b had little or no effect on MCV transduction (Figure S4). One way to reconcile the Lec2 line transduction results with the results observed for neuraminidase-treated A549 (Figure 2) would be to imagine that MCV attachment to a non-sialylated cellular factor allows the capsid to loiter on the cell surface until a hypothetical sialylated entry co-factor is regenerated after removal of the neuraminidase.

### MCV attachment to cultured cells depends on GAGs

**Figure 4:**
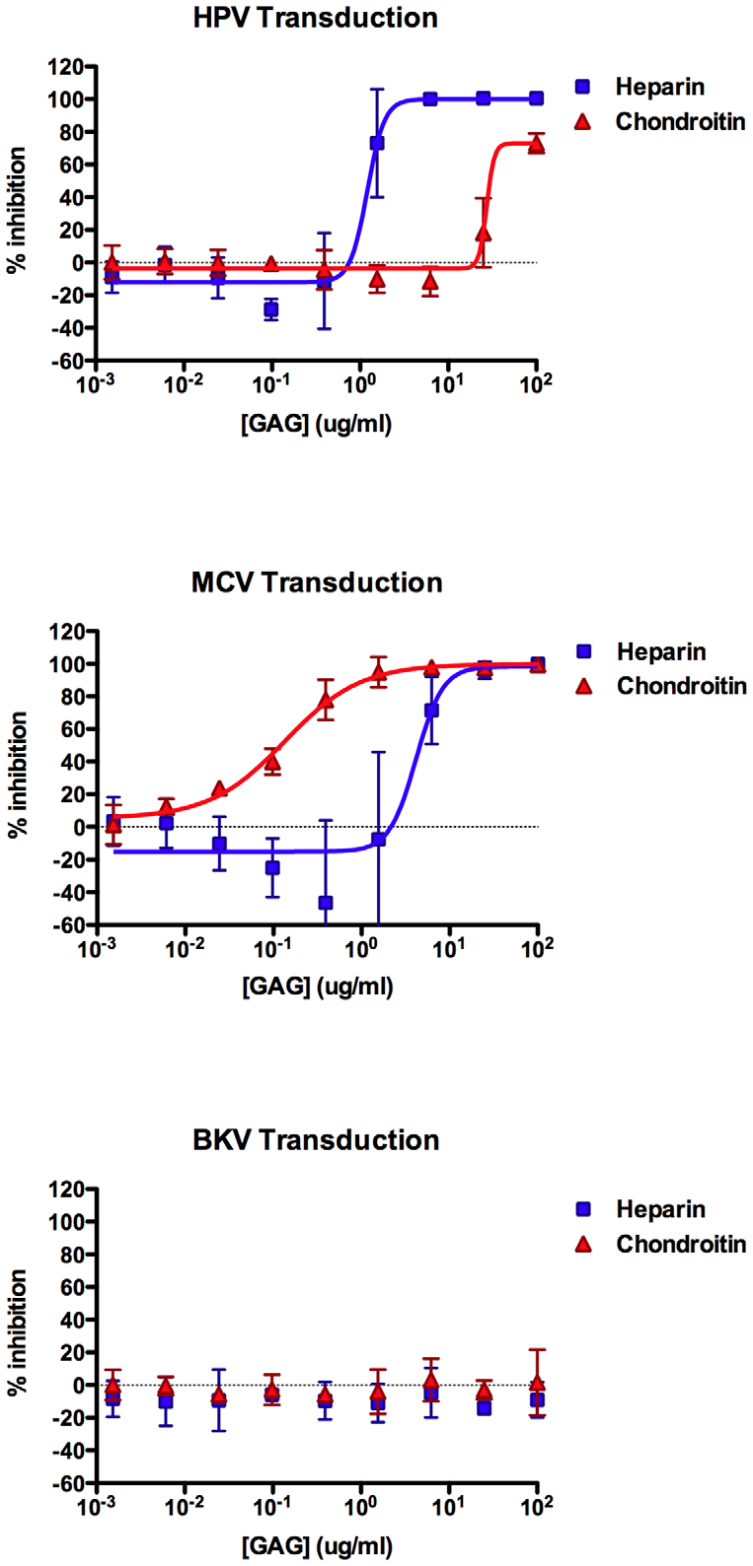
Inhibition of transduction by soluble GAGs. Reporter vector-mediated transduction of A549 cells was measured in the presence of a four fold dilution series of heparin or chondroitin-A/C. The average relative percent inhibition of GFP expression is shown. The curves were fitted using Prism software and error bars represent the standard deviation for three separate experiments.

Our past experience studying HPV binding and entry through interactions with HS led us to test the ability of purified protein-free GAGs, including heparin and chondroitin, to inhibit MCV infection. We found that, heparin can indeed block MCV transduction of A549 cells in a dose-dependent manner, with a 50% effective dose (EC_50_) of 4.2 µg/ml (Figure 4). Interestingly, moderate doses of heparin (∼1 µg/ml) appeared to increase the infectivity of MCV by up to two-fold in some experimental replicates. In contrast to heparin, chondroitin-A/C preparation was a much more effective inhibitor of MCV transduction (EC_50_ = 135 ng/ml) and did not appear to enhance MCV infectivity. Consistent with previous reports [36], [37], the transducivity of an HPV16 reporter vector was blocked more effectively by soluble heparin (EC_50_ = 1.2 µg/ml), while chondroitin-A/C only weakly inhibited HPV transduction. Comparable results were observed for MCV using the kidney-derived neuroblastoid line 293TT [30], [38] (heparin EC_50_≈12 µg/ml, chondroitin-A/C EC_50_≈0.3 µg/ml). The melanoma-derived line SK-MEL-2 and HEKa also showed similar GAG inhibition profiles for MCV reporter vectors (SK-MEL-2 heparin EC_50_≈3 µg/ml, chondroitin-A/C EC_50_≈0.1 µg/ml; HEKa heparin EC_50_≈2 µg/ml, chondroitin-A/C EC_50_≈0.05 µg/ml). As expected, BKV transduction of A549 cells was unaffected by either of the GAG compounds (Figure 4). Other soluble GAGs, such as dermatan sulfate and chondroitin-A, were found to be poor inhibitors of the transduction of all three reporter vectors on A549 cells (data not shown). Since chondroitin-A alone lacked inhibitory efficacy, it is likely that the chondroitin-C (chondroitin-6-sulfate) in the chondroitin-A/C preparation used here was primarily responsible for mediating inhibition of MCV transduction.

Heparin has been shown to inhibit HPV entry by preventing binding of the virus to HS on the cell surface or extracellular matrix [37], [39]. To examine the mechanism through which heparin and chondroitin-C inhibit MCV entry into A549 cells, we measured the binding of Alexa Fluor-labeled MCV, HPV16 or BKV capsids to A549 cells in the presence of increasing concentrations of these GAGs. HPV and MCV binding to A549 cells was inhibited in a dose-dependent manner by both heparin and chondroitin (Figure S5), suggesting that these GAGs inhibit transduction, at least in part, by preventing cell attachment. As expected, heparin and chondroitin had little effect on BKV binding.

**Figure 5:**
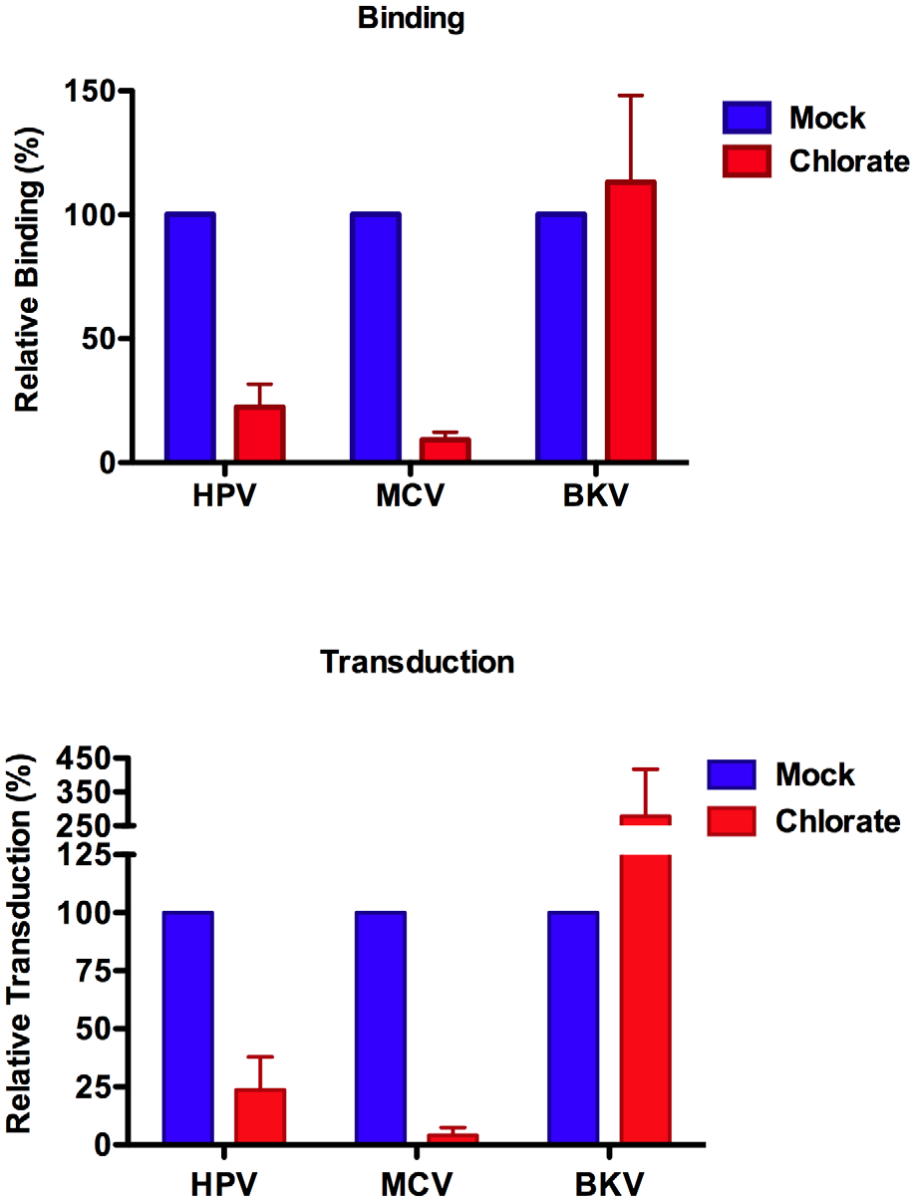
Sulfation is required for MCV binding and entry. A549 cells were propagated for several days in 50 mM sodium chlorate. Binding (top panel) to these cells by Alexa Fluor 488-conjugated capsids was compared to A549 cells cultured without chlorate (mock). Standardized reporter vector-mediated GFP transduction (bottom panel) of A549 cells cultured with or without chlorate. The average of four separate experiments is shown and error bars represent the standard deviation.

Treatment of cell cultures with sodium chlorate inhibits the addition of sulfate groups to GAGs [40], reviewed in [41]. Although chlorate treatment can be toxic to some cell lines (for example, 293TT and HEKa cells do not appear to tolerate 50 mM chlorate), culture of A549 cells in 50 mM chlorate for several weeks did not appear to have noticeable effects on cell morphology or growth rate (data not shown). A549 cells maintained in 50 mM chlorate were extremely resistant to MCV transduction as well as binding (Figure 5). Chlorate-treated A549 cells were likewise resistant to HPV transduction. In contrast, BKV transduction of A549 cells was enhanced by chlorate treatment, confirming that the chlorate-treated cultures were healthy enough to support expression of reporter plasmids delivered via polyomavirus-based vectors. Similar chlorate treatment results were obtained with the melanoma cell line SK-MEL-2 (Figure S6). The data show that sulfate modifications, likely in the form of GAGs, are essential targets of MCV attachment and infectious entry.

**Figure 6:**
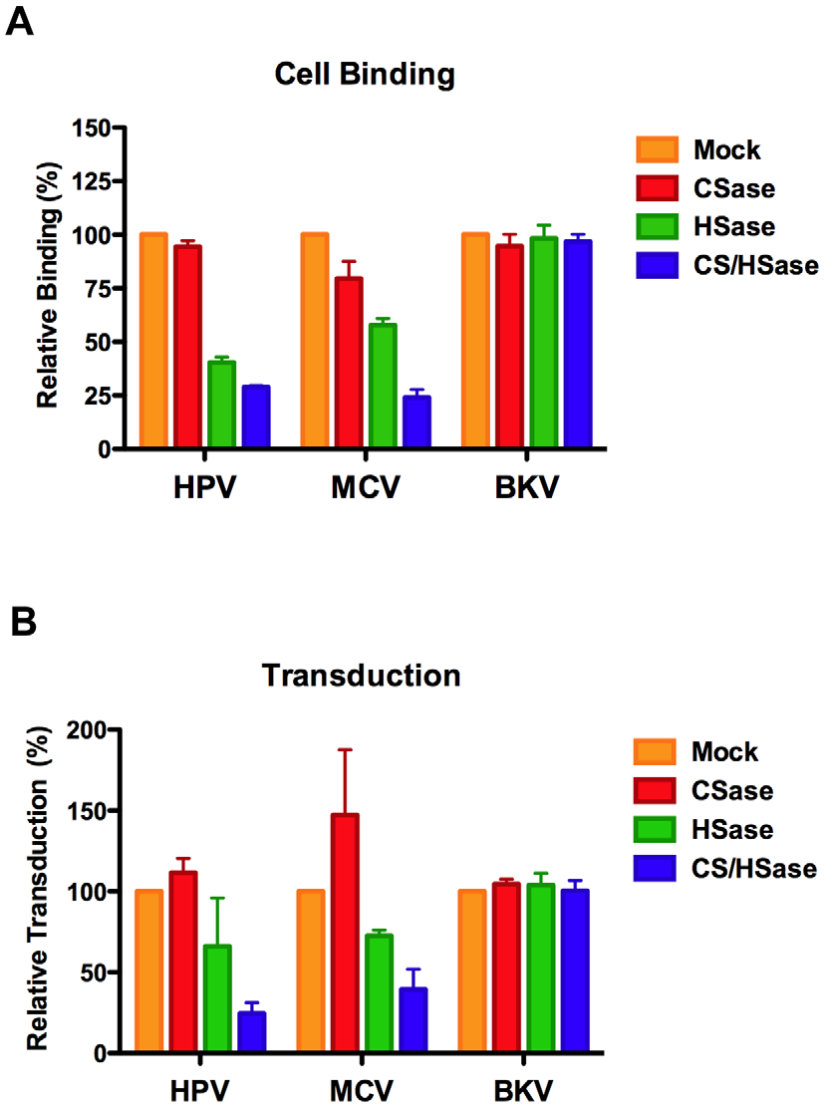
Enzymatic removal of cell surface glycosaminoglycans. A549 cells were treated with chondroitinase ABC (CSase) or with heparinase I/III (HSase), or with both HSase and CSase prior to inoculation with Alexa Fluor 488-conjugated capsids (top panel) or reporter vector (bottom panel). The average of three separate experiments is shown and error bars represent the standard deviation.

To examine the specificity of MCV interaction with different GAG types and to clarify the role of various GAG forms in infectious entry, cell surface HS and/or chondroitin sulfate (CS) were enzymatically removed using heparinase (HSase) and chondroitinase (CSase) enzymes. Enzyme activity and specificity was verified by immunofluorescent staining and flow cytometric analysis of cell surface HS and CS following treatment of A549 cells (Figure S7). Given the superior inhibitory effects of chondroitin-A/C relative to heparin, we expected that CSase treatment would have a greater impact on MCV binding and transduction than HSase treatment. Surprisingly, CSase treatment alone had little effect on MCV binding or transduction, while HSase caused a modest decrease in MCV binding and transduction (Figure 6). Combination HSase/CSase treatments synergistically inhibited MCV binding and transduction. The response of HPV to these treatments was very similar to MCV, while BKV was unaffected.

Combination HSase/CSase treatments were also necessary to effectively inhibit MCV transduction of SK-MEL-2 cells and HEKa cells, confirming the importance of cell surface GAGs for MCV entry into skin-derived cell types (Figure S8). Neither CSase nor HSase alone significantly inhibited or enhanced MCV transduction of SK-MEL-2 or HEKa cells.

### MCV transduction of CHO lines depends on HS but not CS

**Figure 7:**
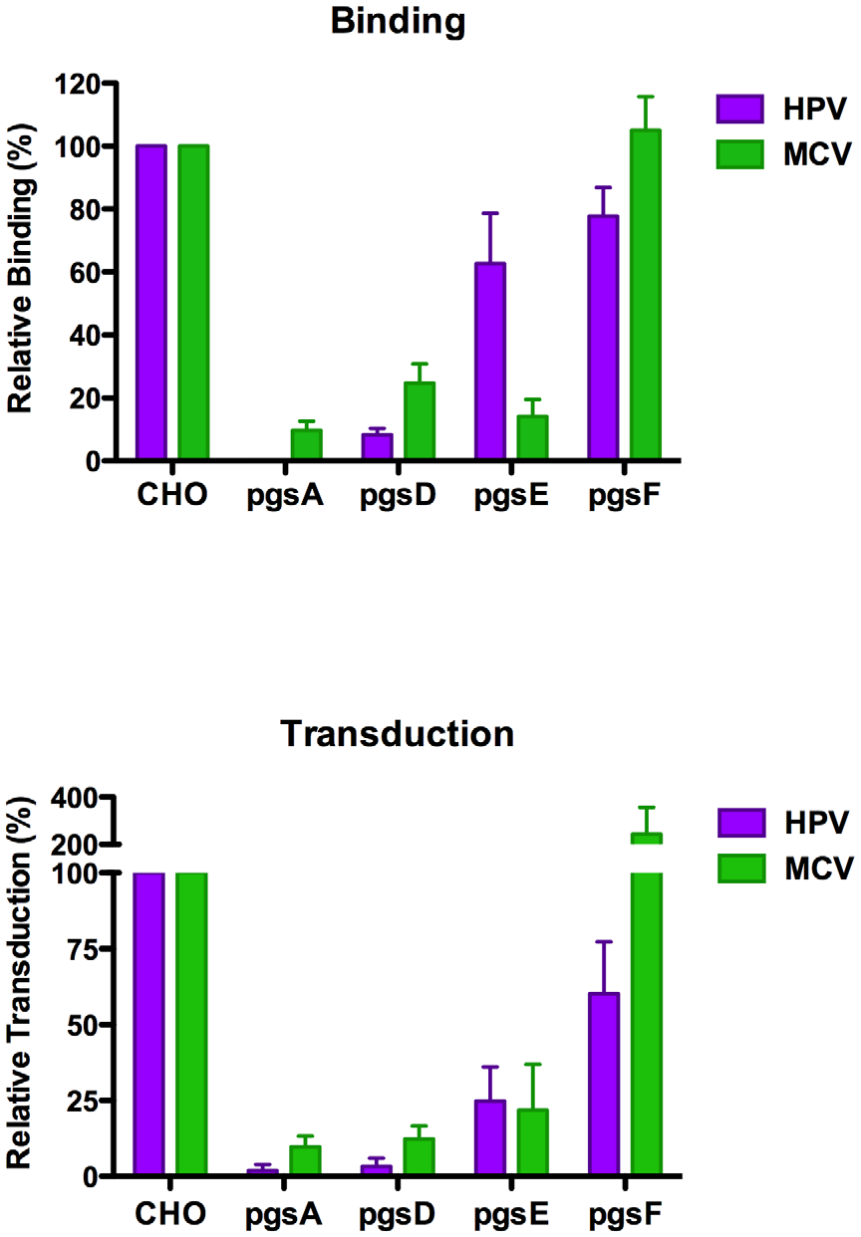
Infection of and binding to GAG-deficient cells. CHO-K1 cells (parental line), pgsA-745 (heparan sulfate (HS) and chondroitin sulfate (CS) deficient), pgsD-677 (HS deficient), pgsE-606 (HS N-sulfate deficient), and pgsF-17 (HS 2-O-sulfate deficient) cells were subjected to a binding assay using Alexa Fluor 488-conjugated capsids (top panel) or transduced with reporter vector (bottom panel). The average of five separate experiments (top panel) or three separate experiments (bottom panel) is shown and error bars represent the standard deviation.

A traditional approach to investigation of the role of particular GAG modifications in viral entry has been to compare the infectability of cell lines carrying mutations in the genes responsible for various steps in GAG biosynthesis [11], [42], [43], [44], [45]. These cell lines range from having no GAGs to simply lacking sulfate or other modifications at specific positions. We found that pgsA-745 cells, which are deficient in both HS and CS, did not bind MCV capsids efficiently and were transduced very poorly in comparison to the parental CHO-K1 line (Figure 7). Similarly, pgsD-677 cells, which lack HS but produce more CS than the parental cells, were highly resistant to MCV transduction. This suggests that HS, and not CS, is of primary functional relevance for MCV-mediated transduction of this cell type.

Two other CHO mutant cell lines are deficient in the biosynthesis of specifically sulfated types of HS. Heparan sulfate modifications occur sequentially and, as a result, disruption of early modification events inhibits downstream modifications as well [11], [46]. Normally, the first step in HS modification involves N-deacetylation and N-sulfation of N-acetylglucosamine (GlcNAc) residues in the HS core chain. A subsequent modification step involves epimerization of glucuronic acid residues to iduronic acid. After these modifications, the HS sequentially becomes an appropriate substrate for 2-O-, 6-O- and 3-O-sulfotransferases. Thus, pgsE-606 cells, which lack GlcNAc N-deacetylase/N-sulfotransferase activity [42], produce HS that is deficient in all forms of modification. Another mutant cell line, pgsF-17, is deficient in 2-O-sulfotransferase function and thus expresses HS that carries N-sulfate and iduronic acid modifications, but lacks 2-O- and 3-O-sulfate modifications [45]. In addition to N-sulfated HS, pgsF-17 cells also produce HS carrying 6-O-sulfate modifications. MCV reporter vectors readily bound and transduced pgsF-17 cells but not pgsE-606 cells, (Figure 7) indicating that HS epimerization, N-sulfation and/or 6-O-sulfation are required to support MCV-mediated transduction, while HS 2-O- and 3-O-sulfation are dispensable. In comparison to HPV16, we found that the GAG type preferences of the two reporter vectors differ somewhat, as pgsF-17 cells show reduced HPV transduction, while the MCV reporter vector readily transduced this line. This result is consistent with previous reports indicating that 2-O-sulfate groups on HS are required for efficient transduction of cultured cells with HPV16 vectors [47].

**Figure 8:**
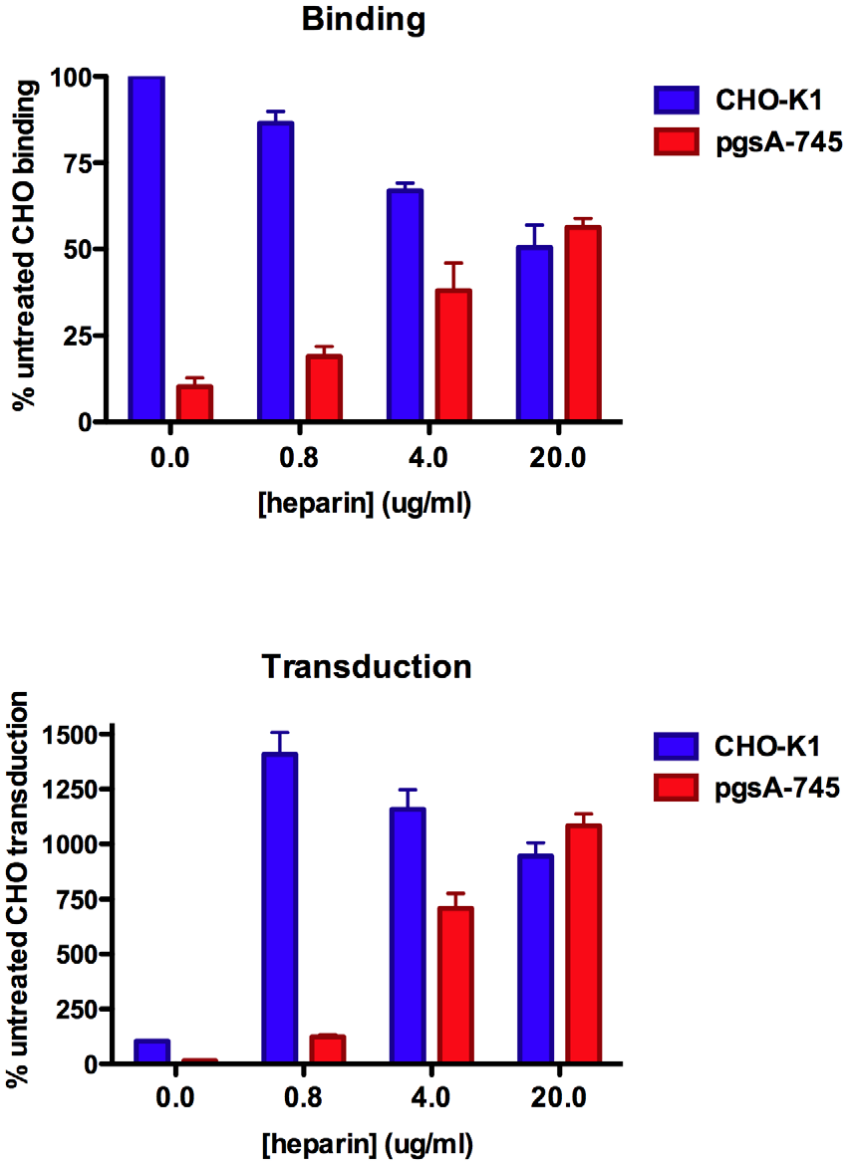
Enhancement of MCV binding and infection by exogenous heparin. CHO-K1 cells (parental line) or pgsA-745 cells (HS and CS deficient) were used to examine binding of Alexa Fluor 488-conjugated MCV capsids in the presence of the indicated concentration of heparin (top panel). Fluorescent intensity of the cells was standardized to CHO-K1 cells incubated with conjugated capsids in the absence of heparin. The average of three separate experiments is shown and error bars represent the standard deviation. CHO-K1 and pgsA-745 cells were plated, and six hours later treated with the indicated dose of heparin and MCV reporter vector (bottom panel). The percent of cells GFP+ 72 hours after inoculation was standardized to CHO-K1 cells incubated with reporter vector in the absence of heparin. The extent of the observed enhancement mediated by heparin varied from one experiment to the next and depended on the level of infection achieved in the heparin-untreated culture. However, the trend was always the same in five independent experimental repeats. A representative experiment performed in triplicate is shown with error bars representing the standard deviation.

Because many cell surface proteins display GAG-binding motifs, most cell types have substantial capacity to bind free GAGs non-covalently [48]. Non-covalently associated GAG chains, including exogenously-provided heparin, can participate in a wide variety of biological functions. For example, free heparin can serve as a functional “bridge” between vascular endothelial growth factor 164 and its co-receptor neuropilin 1 [49], [50]. Consistent with this type of bridging effect, we found that provision of exogenous heparin increased the transducibility of GAG-deficient pgsA-745 cells in a dose-dependent manner. At an apparent optimal concentration of heparin in the media of around 20 µg/ml, pgsA-745 cells became 10-fold more transducible than untreated parental CHO-K1 cells (Figure 8). MCV-mediated transduction of CHO-K1 cells was also enhanced by exogenous heparin, but the most effective dose was lower, presumably reflecting a reduced need for exogenous heparin due to the presence of native GAGs. Transduction of pgsD-677 and pgsE-606 cells was similarly enhanced by exogenously-supplied heparin, confirming that a heparin-like GAG is the primary missing factor required for MCV-mediated transduction of these HS modification mutant CHO lines (data not shown). Moderate doses of chondroitin-A/C also increased MCV transduction of GAG-deficient cells slightly, but the effect was very small in comparison to the effect of heparin (data not shown). In contrast to the GAG mutant CHO cell lines, MCV transduction of Lec2 cells was not rescued by exogenously-supplied heparin, suggesting that the block to MCV transduction in Lec2 cells occurs downstream of HS binding (data not shown).

The rescue of MCV transduction of pgsA-745 cells by exogenous heparin correlated with an improvement in capsid binding to the cell (Figure 8). Surprisingly, neither pre-incubation of pgsA-745 cells with heparin nor pre-incubation of reporter vector stocks with heparin showed dramatic effects on MCV binding or transduction (data not shown). A possible explanation for this finding might be that one or more interactions in a hypothetical termolecular complex between heparin, MCV and cell surface binding targets may be of low overall affinity and relatively transitory. To test the idea that limitation of the ability of MCV to loiter on the cell surface might curtail access to a hypothetical sialylated co-receptor, we performed MCV binding and transduction assays on pgsA-745 cells supplied with exogenous heparin and treated with or without neuraminidase. Although neuraminidase treatment again had no effect on MCV binding in these experiments, the treatment modestly suppressed MCV transduction (Figure S9). The results are consistent with the idea that limitation of the ability of MCV to loiter on the cell surface reduces the engagement of a sialylated entry co-factor that regenerates after neuraminidase treatment.

### Analysis of MCV interaction with immobilized HS

**Figure 9:**
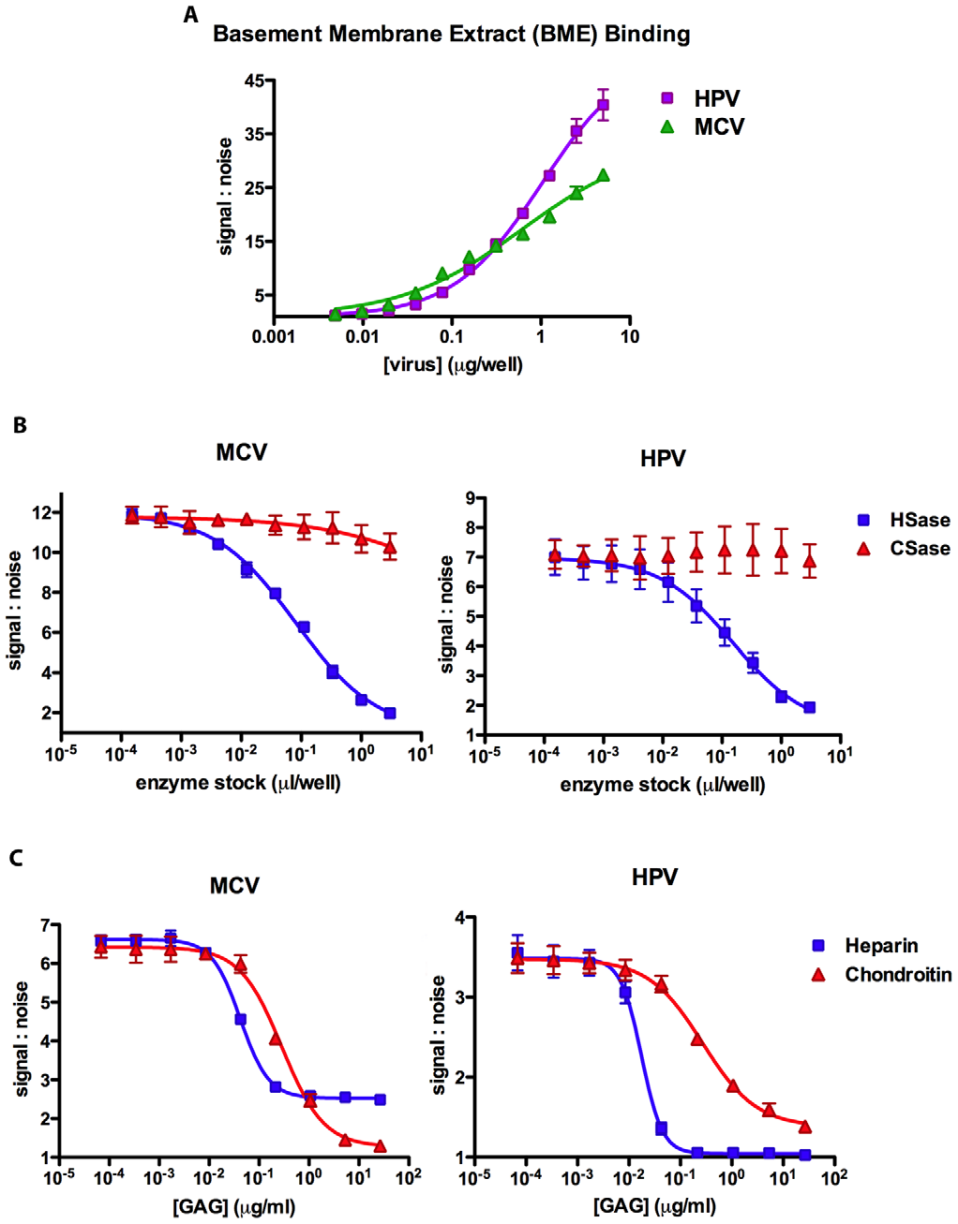
Kinetics of glycosaminoglycan binding. Microtiter plates were coated with GAG-rich basement membrane extract (BME) and (A) used to examine MCV and HPV capsid affinity by varying the dose (two fold) of VP1 added to wells. (B) BME coated plates were incubated with increasing concentrations (three fold) of heparinase I/III (“HSase”) or chondroitinase ABC (“CSase”) prior to adding a single dose of capsids to each well. (C) Heparin or chondroitin-A/C were serially diluted (five fold) in buffer containing capsids prior to analysis of BME binding. The amount of capsid bound to BME was determined by PicoGreen fluorescence detection of encapsidated DNA. The average of two (A), three (B) or four (C) replicates is shown. The curves were fitted using Prism software and error bars represent the standard deviation.

The results shown above suggest that attachment to cell surface HS is a critical step in MCV vector-mediated reporter gene transduction. However, the strong inhibition of MCV entry by soluble chondroitin-A/C raises questions surrounding the precise interaction of MCV with different GAG types. In an effort to better understand the physical interaction between MCV and GAGs, an ELISA-style binding assay was developed using a commercially available GAG-rich basement membrane extract (BME) derived from murine Engelbreth-Holm-Swarm tumor to coat the surface of 96-well protein-binding plates. Since the binding of VP1-specific antibodies might be affected by GAG-capsid interactions, we elected to detect bound reporter vector particles using Quant-iT PicoGreen stain [51] to render encapsidated DNA carried within the particles fluorescent. Increasing concentrations of HPV16 or MCV capsids in the BME-coated wells correlated with an increase in fluorescence (Figure 9A). BKV capsids bound the BME-coated wells very poorly (data not shown), suggesting the BME displays few binding sites for BKV.

To determine whether capsid binding to the BME was the result of interactions with GAGs, BME-coated wells were pre-treated with increasing doses of HSase or CSase. Only HSase treatment of the BME resulted in major dose-dependent decreases in binding by MCV and HPV, and the highest concentration of HSase resulted in nearly complete abrogation of binding (Figure 9B), indicating that both viruses predominantly bind HS displayed on BME.

The slope of the MCV capsid dose-response curve for binding to BME (Figure 9A) is relatively shallow, with a Hill coefficient of 0.64±0.14. A simple explanation for the occurrence of Hill slopes of less than one is that the assay is simultaneously measuring multiple binding interactions with differing affinities. This explanation is consistent with the fact that native GAGs are heterogenous and carry complex modifications that can dramatically alter their affinity for GAG-binding proteins. To circumvent this problem, we measured the ability of the more homogenous preparations of heparin and chrondroitin-A/C to interfere with the binding of MCV to BME. Interestingly, although high doses of chondroitin-A/C were able to entirely block the binding of MCV capsids to the BME, apparently saturating doses of heparin reduced MCV binding by only about 75% (Figure 9C). A model for these observations would be that the BME displays two distinct targets for MCV binding and the capsid carries two distinct glycan-binding motifs. Under this model, chondroitin-A/C is capable of blocking both of the glycan-binding motifs on the capsid surface, while heparin is capable of blocking only one binding motif.

### Confirmation using a native MCV infection assay

Systems for culturing MCV have not yet been developed. We have previously speculated that the relative inactivity of recombinant MCV genomes transfected into cultured cells may reflect regulation of the viral life cycle in a manner reminiscent of the extensive regulatory controls on the papillomavirus life cycle [17], [52]. Although we have previously shown that the genomic DNA of MCV primary isolates can drive the production of low levels of native virions after transfection into 293TT cells, the yield of native virions was relatively poor [17]. We found that virion yield can be improved substantially if the cloned genome is co-transfected together with expression plasmids encoding MCV small and large T antigen cDNAs (data not shown). To monitor the infectivity of native MCV virions, we generated a 293TT-based line, named 293-4T, which stably expresses the MCV small and large T antigen proteins. The stable line supports the replication of MCV genomes delivered by infection with native MCV virions, allowing monitoring of the infection using quantitative PCR (qPCR). The extent of MCV replication observed over several days varied between experiments, ranging from 7.5 fold to 70 fold. In separate experiments, we found that purified native MCV virions can be propagated in 293-4T cells (Figure S10).

**Figure 10:**
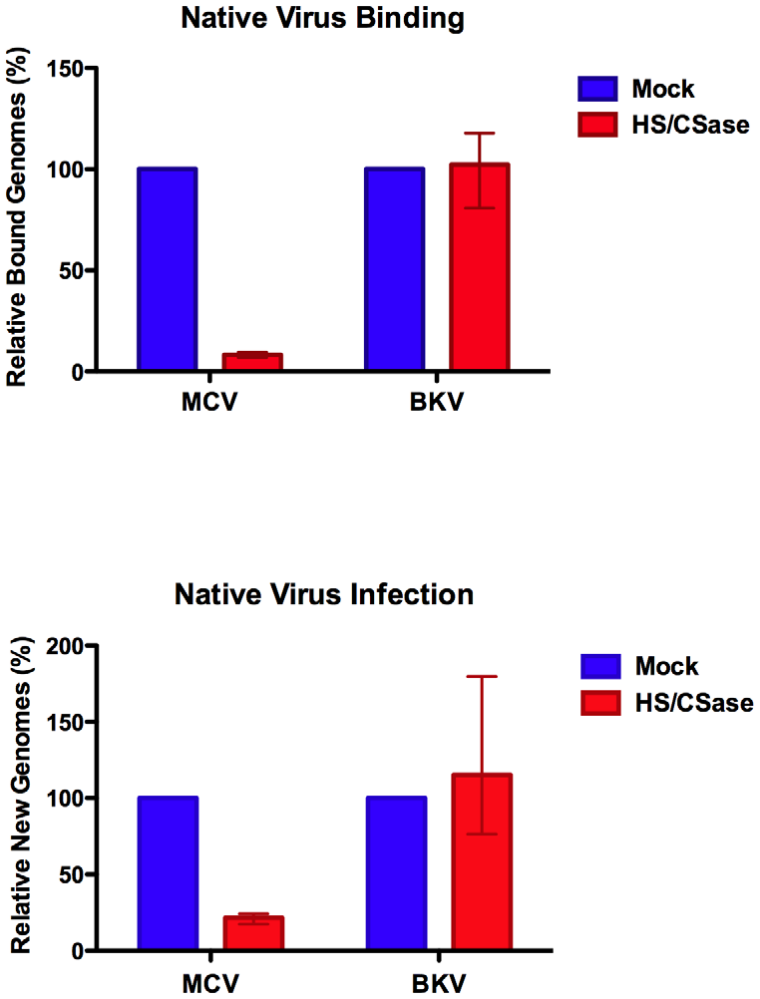
Native MCV binding and infection of cells treated with heparinase and chondroitinase. 293-4T cells were treated with chondroitinase ABC and heparinase I/III (HS/CSase) or mock treated prior to the addition of native MCV or BKV virions. The number of copies of cell-associated MCV or BKV DNA was measured by qPCR 45 minutes after inoculation or 5–6 days later. The percent of bound or replicated genome copies relative to mock treatment in three separate experiments is shown. Error bars represent the range of observed values.

Using the native virion/293-4T infection system and enzymatic removal of cell surface GAGs, we confirmed that GAGs are required for MCV infection. qPCR analysis of 293-4T cells harvested immediately after viral inoculation and washing of cells treated with or without HSase/CSase revealed a 90–93% reduction in the number of cell-bound virions in the HSase/CSase treatment condition (Figure 10). The failure of the virus to bind efficiently to the HSase/CSase treated cells was reflected by a comparable decrease (76–83%) in the number of replicated viral genomes observed after 5–6 days of cell growth. To control for cell health after enzyme treatment, parallel experiments were performed to measure the number of genome copies for native BKV virions. As expected, native BKV infection was either unaffected by HSase/CSase treatment or, in one of the three replicates, modestly enhanced by the enzyme treatment.

Native MCV virions were also used for a panel of additional confirmatory experiments (data not shown) on cell types that do not appear to support the replication of MCV genomes delivered by native virions. For these experiments we made the simplifying assumption that failure to bind the cell would result in failure to infect the cell. Native virion binding was measured using qPCR of viral genomes stably associated with cells. In an initial control experiment, we found that monoclonal antibodies specific for assembled MCV capsids [53] blocked the binding of MCV virions to A549 cells. Native MCV virions also failed to bind A549 cells in the presence of chondroitin-A/C. Treatment of A549 cells with sodium chlorate likewise prevented the binding of native MCV virions. We also found that native MCV virions readily bind both Lec2 and Lec2-mslc, confirming that native MCV does not require sialylated carbohydrates for attachment to cells. Taken together, the data show that native MCV virions exhibit binding and infectivity characteristics similar to MCV reporter vectors.

## Discussion

Interaction with cell surface receptors is an essential first step in the process of viral infectious entry. Here we present multiple lines of evidence demonstrating that the initial attachment of MCV to cultured cells is mediated primarily by GAGs. Like HPV16, MCV binding and infectious entry can be antagonized by soluble GAGs and the attachment and infectivity of both viruses depends on the presence of cell surface GAGs. Although MCV capsids can bind to both CS and HS, experiments using CHO-based mutant cell lines indicate that N-sulfated and/or 6-O-sulfated forms of HS are specifically required for infectious entry. The handful of other polyomaviruses whose infectious entry pathways have been carefully studied all appear to utilize sialic acid-containing receptors for the initial cell attachment step of the infectious entry process [24], [54], [55], [56], [57]. Our studies show that sialylated glycans are not required for initial attachment of MCV to cultured cell lines. Further work is needed to determine whether MCV is unusual in this regard or rather provides an example of a common trait among the two dozen or so polyomavirus species that have not yet been subjected to extensive scrutiny.

MCV appears to require a sialylated glycan for a post-attachment step in the infectious entry process. It remains uncertain whether this apparent requirement for sialylated glycans is due to indirect or direct effects. For example, failure to sialylate a cellular factor might impair a biological function or subcellular localization required to support MCV entry. In this scenario, MCV might not directly bind the sialylated glycan. A more intriguing possibility is that MCV directly interacts with a sialylated glycan during the infectious entry process. Although we found no clear evidence for direct interactions between MCV capsids and sialic acid residues on cultured human cell lines, Erickson and colleagues have previously shown that MCV VP1 capsomers can bind neuraminidase-sensitive factors in concentrated extracts of sheep RBCs [23]. Erickson and colleagues also demonstrated that MCV VP1 can bind GT1b when the ganglioside is presented at high concentrations in a cell-free flotation system. This suggests a possible scenario in which an unknown sialylated factor that resembles the glycan headgroup of GT1b serves as a co-receptor that the virion directly engages after initial attachment to the cell via HS. This would be analogous to the infectious entry of HIV, which generally requires the direct engagement of a chemokine co-receptor after initial attachment to a primary attachment receptor, CD4. It is tempting to speculate that the hypothetical sialylated co-receptor required for MCV entry might be a ganglioside. However, the fact that CHO-based lines, which are deficient in complex gangliosides [34], [35], are readily transducible by MCV reporter vectors would argue against this hypothesis. Further work is needed to determine which sialylated glycans, if any, MCV binds during infectious entry into human cells.

Although our results using CHO cell lines indicate that HS is a more important factor than CS for MCV infectious entry, soluble heparin proved to be a less effective inhibitor of entry than chondroitin-C on all tested cell lines, including CHO (Figure 4). The results of the competitive inhibition experiments on basement membrane extracts (Figure 9) suggest a possible explanation for the apparently greater efficacy of chondroitin-C for inhibiting MCV infection. These analyses indicate that although MCV has higher affinity for heparin, chondroitin-C may be a better infection inhibitor because it blocks a secondary glycan binding site on the MCV virion surface that the highly homogenous heparin preparation cannot saturate. This model could explain the observation that chondroitin-A/C is a more effective inhibitor of MCV transduction.

Although heparin doses >10 µg/ml effectively inhibited MCV transduction of several human cell lines, lower doses of heparin showed variable enhancement of MCV transduction of these lines (Figure 4 and data not shown). For CHO-based lines, heparin only enhanced infectivity, even at 20 µg/ml doses (Figure 8). The variable ability of heparin to either inhibit or enhance infectivity on various cell types is reminiscent of models for antibody-dependent neutralization or enhancement of the infectivity of flaviviruses (reviewed in [58]). In this model, antibodies that can neutralize flaviviruses when bound at high occupancy can also enhance infection when bound at low occupancy. It is thought that this effect reflects the ability of some antibodies to serve as a bridge between the partially occluded virion and antibody-Fc receptors expressed on the surface of some cell types. Analogously, heparin might serve as a bridge in a termolecular complex between heparin-binding proteins on the cell surface and heparin binding motifs on the surface of the MCV capsid. A similar model has recently been proposed for the infectious entry of human T-cell leukemia virus-1 (HTLV-1) [59]. It is also conceivable that, rather than forming a physical bridge between the MCV capsid and cell surface GAG-binding factors, heparin might induce a reversible change in the capsid structure that, in turn, permits direct binding of the capsid to a cellular co-receptor moiety. This would be reminiscent of conformational changes that are thought to occur in HPV capsids during infectious entry (reviewed in [60]). In either event, it is clear that the effectiveness of GAG inhibition of MCV reporter vectors can vary dramatically between cell lines. Resolution of this issue will require more detailed knowledge of the cellular factors that support the post-attachment steps of MCV infectious entry.

Polyomaviruses have a long and complex history as suspected agents of human cancer [61]. The data implicating MCV as a cause of cancer in epidermal Merkel cells appears to be the strongest case yet described for a polyomavirus. That MCV particles can be isolated from human skin surfaces and cause tumors is reminiscent of certain aspects of HPV biology. Our data clearly show that both of these viruses require initial attachment to specific forms of HS, followed by transfer to poorly understood co-receptors for infectious entry to occur. Whether this is coincidence is difficult to determine, but it will be interesting to learn if these unrelated viruses share other aspects of their biology.

## Methods

### Cells and plasmids

A549 cells and SK-MEL-2 cells were obtained from the Developmental Therapeutics Program (NCI/NIH) and maintained in RPMI medium (Invitrogen) supplemented with 5% FBS (Sigma) and Glutamax-I (Invitrogen). HEKa (human epidermal keratinocytes, adult) were purchased from Invitrogen and maintained in Medium 254 supplemented with HKGS. CHO-K1 cells, pgsA-745, pgsD-677, pgsE-606, Pro5, and Lec2 cells were obtained from ATCC and maintained in DMEM (Invitrogen) with 10% FBS, Glutamax-I and MEM non-essential amino acids (D10 medium). pgsF-17 cells (a kind gift from Jeff Esko [45]) were maintained in D10 medium. Medium for the Lec2-mslc cells was supplemented with blasticidin S (5 µg/ml; Invitrogen). 293TT cells were maintained in D10 supplemented with hygromycin (250 µg/ml; Roche) and 293-4T were maintained in D10 supplemented with zeocin (100 µg/ml; Invitrogen) and blasticidin S (5 µg/ml; Invitrogen).

Plasmids reported in this study will be made available through Addgene.org. The pMslc plasmid used to restore expression of SLC35A1 (accession number NM_006416) in Lec2 cells was created by transferring the human cDNA clone of SLC35A1 (OriGene, restriction enzymes XbaI and NcoI) into the expression cassette of pMONO-blasti-msc (InvivoGen, restriction enzymes AvrII and NcoI). 293-4T cells were created through two stable transfection steps. In the first step, 293TT cells were transfected with pMtB, an expression plasmid carrying the small T antigen ORF of MCV isolate R17a (GenBank accession number HM011555, [17]) in the expression cassette of pMONO-blasti-msc. Stable blasticidin-resistant clones were isolated by limiting dilution and analyzed for small T antigen expression by immunofluorescence microscopy and western blot using polyclonal serum raised against bacterially-produced MCV small t antigen fused to a maltose binding protein affinity tag (unpublished data). Stable expression of MCV small t antigen appears to be relatively toxic to 293TT cells and few clones maintained expression of the protein. One clone that stably expressed MCV small t antigen was super-transfected with a construct named pADL*, encoding MCV Large T antigen. The construct was generated by first silently mutating the splice donor and acceptor sites for the 57 kT isoform of MCV Large T antigen in the context of expression plasmid pCDNAclt206antigen1 (p2582), which was a generous gift from the Chang/Moore lab [62]. The Large T antigen ORF was also modified to remove the V5 epitope tag and proline residue 156 was mutated to serine to match the wild-type MCV consensus at that site. The modified T antigen gene was transferred into pMONO-zeo-mcs (InvivoGen) by restriction enzyme-based cloning. The polyclonal pADL* population was selected with both zeocin and blasticidin and the resulting stable line was named 293-4T. Nucleotide maps of plasmids used in this work and detailed protocols are available on our laboratory website <http://home.ccr.cancer.gov/Lco/>.

### Reporter vector production and purification

MCV reporter vector stocks were produced using previously reported methods [25], [63]. Briefly, 293TT cells [30] were transfected with plasmids pwM2m [53] and ph2m [25] expressing codon-modified versions of the VP1 and VP2 genes of MCV strain 339. HPV16 reporter vectors were produced using the L1/L2 expression plasmid p16sheLL [36]. Production of BKV reporter vectors used a mixture of four novel plasmids, pwB2b pwB3b, ph2b and ph3b, which carry codon-modified versions of the capsid proteins of BKV genotype IV isolate A-66H (accession number AB369093, [64]). The capsid protein expression plasmids were co-transfected with a mixture of two reporter plasmids, pYafw [30] and pEGFP-N1 (Clontech) which express GFP from recombinant EF1α or CMV immediate early promoters, respectively. Forty-eight hours after transfection, the cells were harvested and lysed in Dulbecco's phosphate buffered saline (DPBS, Invitrogen) supplemented with 9.5 mM MgCl_2_, 25 mM ammonium sulfate (starting from a 1 M stock solution adjusted to pH 9), antibiotic-antimycotic (Invitrogen), 0.5% Triton X-100 (Pierce) and 0.1% RNase A/T1 cocktail (Ambion). The cell lysate was incubated at 37°C overnight with the goal of promoting capsid maturation [65]. Lysates containing mature capsids were clarified by centrifugation for 10 min at 5000×g twice. The clarified supernatant was loaded onto a 27–33–39% iodixanol (Optiprep, Sigma) step gradient prepared in DPBS with a total of 0.8 M NaCl. The gradients were ultracentrifuged 3.5 hours in an SW55 rotor at 50,000 rpm (234,000×g). Gradient fractions were screened for the presence of encapsidated DNA using Quant-iT Picogreen dsDNA Reagent (Invitrogen). The VP1 concentration of Optiprep-purified reporter vectors was determined by comparison to bovine serum albumin standards in SYPRO Ruby (Invitrogen)-stained SDS-PAGE gels. The MCV reporter vector stock contained 8.6 ng of VP1/µl, the BKV vector stock contained 4.3 ng of VP1/µl, and the HPV vector stock contained 2.9 ng of L1/µl. In various experiments examining reporter vector-mediated transduction, 0.2–0.4 µl of MCV stock, 0.3–0.6 µl of BKV stock, and 0.03–0.15 µl HPV stock was used per 96 well plate well. These concentrations generally produced between 5 and 25% GFP positivity in cell populations at the time of flow cytometric analysis.

Recombinant capsids were produced as above, except that Benzonase (Sigma) and Plasmid Safe (Epicentre) nucleases were added to the lysis buffer at 0.1% each, with the goal of liberating capsids carrying fragments of cellular DNA [63]. Hemagglutination and basement membrane extract experiments used unlabeled capsids, while cell-binding studies used capsids covalently conjugated to Alexa Fluor 488 using previously-reported methods [53]. For production of Alexa Fluor 488 labeled capsids, a reporter plasmid encoding Gaussia luciferase (phGluc; [25]) was included in the initial transfection mixture. All conjugated capsid stocks were between 150 and 275 ng/µl and binding experiments used 0.2–0.4 µl of stock per 5×10^4^ cells suspended in a volume of 100 µl. This generally achieved 10–30 fold fluorescence over background in flow cytometric analyses.

### Hemagglutination assays

Sheep blood in sodium citrate was purchased from Lampire Biological Products. Human type O+ blood was collected by finger prick immediately prior to use. Red blood cells (RBCs) were washed and suspended in PBS without calcium or magnesium (Invitrogen) at a final concentration of 0.5% (v/v). The suspension was chilled on ice in round-bottom 96-well plates then mixed with various doses of purified capsids and allowed to settle overnight at 4°C.

### Reporter vector (pseudovirus) based infectivity studies

A549 cells were plated at 7,500 cells/well in 50 µl of culture medium in a 96 well plate the day prior to infection. Stock solutions of porcine heparin (Sigma H4784), porcine dermatan sulfate (chondroitin sulfate B, Sigma C3788), bovine chondroitin sulfate-A (Sigma C9819), or shark chondroitin sulfate-A/C (Sigma C4384) were dissolved at 10 mg/ml in PBS (Invitrogen). The GAGs were serially diluted in media to 3× the indicated concentration and 50 µl was added to cells. Reporter vector stock was then added to the cells+GAG mixture in a volume of 50 µl. To minimize plate edge effects, the outer wells of the plate were not used for the assay and were instead filled with culture medium. Approximately 72 hrs post-infection, cells were incubated with trypsin to detach them from the plate and transferred to an untreated 96 well plate and suspended in wash medium (WM; DPBS with 1% FBS, antibiotic-antimycotic, and 10 mM HEPES, pH 8) and analyzed by flow cytometery for GFP reporter gene expression in a FACS Canto II with HTS (BD Biosciences).

To calculate 50% effective inhibitory concentrations (EC_50_), Prism software (GraphPad) was used to fit a variable slope sigmoidal dose-response curve to values representing the percentage of GFP positive cells relative to untreated infected cells. Error bars represent the standard deviation for at least three independent experiments.

### Binding to cells in the presence of GAGs

Cells were dislodged using PBS supplemented with 10 mM EDTA, and then pipetted with an equal volume of WM. Fifty thousand cells were added to wells of an untreated 96 well plate and washed once with WM. Cells were then washed once with a dilution series of GAG in WM. Next, the same dilution series of heparin or chondroitin A/C in WM containing Alexa Fluor conjugated capsids was added to cells, such that each well contained about 60 ng of VP1 in the indicated concentration of GAG. These plates were incubated at 4°C for one hour, and then cells were washed 3 times in WM before measurement of their fluorescence by flow cytometry.

### Transduction of sodium chlorate treated cells

A549 cells were cultured in D10 supplemented with 50 mM sodium chlorate (Sigma) for 2–6 days, then pre-plated overnight at 9,000 cells/well in 96 well plates. The next morning, half the plate was changed into medium without chlorate to allow regeneration of sulfate modifications. The other half of the plate was changed into fresh chlorate-containing media. Six to eight hours later, reporter virus was added in medium with or without sodium chlorate to maintain the concentration of chlorate present on the cells. Forty-eight hours later, the cells were fed by addition of 100 µl of media without chlorate. After a total of about 72 hours, cells were harvested for analysis of GFP expression by flow cytometry.

### Enzymatic Removal of cell-surface sialic acids or GAGs

For experiments examining the effects of neuraminidase treatment on reporter vector transduction, cells pre-plated in 96 well plates were washed and incubated with 50 µl of DPBS containing 70 mU of neuraminidase from *Arthrobacter ureafaciens* (NorthStar Bioproducts) per 5×10^5^ cells for 1 hour at 37°C. The cultures were then inoculated with reporter vector stock and incubated for an additional 2 hours at 37°C. The cultures were then washed once and fed with 100 µl of culture medium. In some replicates, culture medium was added directly to the neuraminidase-containing PBS in the culture well. Removing or washing away the neuraminidase/reporter vector mixture did not appear to alter the experimental outcome. After three days, the cells were harvested and analyzed for GFP expression by flow cytometry. For binding studies, conjugated capsids were added to 5×10^4^ neuraminidase-treated (or mock-treated) cells in suspension in an untreated 96 well plate and incubated for 1 hour at 37°C. The cells were then washed three times prior to analysis of fluorescence by flow cytometry.

Heparinase I (50 units, Sigma) and heparinase III (5 units, Sigma) were solubilized in 100 µl each of resuspension buffer containing 20 mM Tris, pH 7.5, 50 mM NaCl, 4 mM CaCl_2_ and 0.01% BSA. The two enzymes were then combined. Chondroitinase ABC (2 units, Sigma) was solubilized in 200 µl of resuspension buffer. A549 cells plated the day prior at 7,500 cells/well in a 96 well plate, were washed once with digestion buffer (20 mM HEPES, pH 7.5, 150 mM NaCl, 4 mM CaCl_2_ and 0.1% BSA), and then treated with 2.5 µl of heparinase I/III stock, 2.5 µl of chondroitinase stock (or both) in 50 µl of digestion buffer. Cells were incubated in digestion buffer with or without enzyme for 2 hours at 37°C. Various doses of reporter vector stock were then added to the wells in 50 µl of OptiMEM-I (Invitrogen) and incubated for an additional 1 hour at 37°C. The cells were washed twice with culture medium, and then incubated in 150 µl/well culture medium for three days. Cells were then analyzed for GFP expression by flow cytometry, as above. For binding analyses, 2×10^5^ cells dislodged using PBS and 10 mM EDTA, were treated with 3.5 µl each enzyme in digestion buffer for 1.5 hours at 37°C. Alexa Fluor conjugated capsids diluted in Opti-MEM were then added to the cells and incubated for an additional one hour at 37°C prior to washing and flow cytometric analysis.

### Comparison of CHO-K1 and GAG mutant cell infection and effect of exogenous GAGs

CHO-K1, pgsA-745, pgsD-677, pgsE-606, and pgsF-17 cells were plated at a density of 10,000 cells/well in 50 µl culture medium in a 96 well plate. Binding and infectivity studies to analyze the effect of exogenous GAGs were performed as above, except that cells were pre-plated and infected the same day in order to avoid changes in cell number resulting from slightly differing rates of growth.

### Basement membrane extract (BME) binding assay

Cultrex BME PathClear (Trevigen #3432-005-02), a BME preparation derived from murine Engelbreth-Holm-Swarm tumor, was aliquoted and stored according to manufacturer's instructions. Black Microfluor 2 ELISA plates (Thermo) were coated overnight with 1 µg of BME per well in a volume of 150 µl. Coated plates were emptied and treated with 200 µl/well of 1× Blocker BSA (Pierce) in PBS. The block was incubated for 2 hours, with rocking, at room temperature. The plate was then washed twice with PBS plus 0.05% Tween 20 (PBS/Tween; BioRad).

To examine direct virus binding to BME, a two-fold dilution series of HPV or MCV capsids beginning at 5 µg of VP1/well in 150 µl PBS/Tween was examined for binding to BME-coated plates. Binding reactions were conducted for two hours at room temperature, with rocking. To analyze the binding of capsids to BME for all experiments, the plate was washed three times with PBS/Tween, and then treated with 150 µl/well Quant-iT PicoGreen dsDNA Reagent (Invitrogen) in TE buffer supplemented with 0.1% Proteinase K stock (Qiagen). The plate was incubated in a 65°C oven for 1 hour, and then cooled for 15 min at room temperature in the dark before measuring fluorescence in a BMG Labtech POLARstar Optima microplate reader.

To analyze the effect of enzymatic cleavage of GAGs on virus binding, a three fold dilution series of heparinase or chondroitinase (prepared as described above in the section on enzymatic removal of cell-surface sialic acids or GAGs) beginning with 4.5 µl of enzyme stock per well in 150 µl of digestion buffer was added to the prepared plate and incubated for 2 hours at 37°C. The plates were then washed twice with PBS/Tween, and 100 ng/well of capsids in 150 µl of PBS/Tween was added to all treated and mock-treated control wells.

To measure competitive inhibition of capsid binding with heparin and chondroitin A/C, a five fold dilution series of each GAG, beginning with 100 µg of GAG per well was mixed with 50 ng of capsids in 150 µl of PBS/Tween, and then added to the BME-coated plate.

### Native MCV production and purification

MCV virions were produced by co-transfecting 293TT cells [30] with recombinant MCV isolate R17a genomic DNA, reconstituted by intramolecular re-ligation at 4 µg of plasmid DNA per ml using T4 DNA ligase (NEB). The re-ligated MCV genomic DNA was co-transfected with expression plasmids carrying the MCV Large T (pADL*) and small t (pMtB) antigen genes. Cells were expanded for five days after transfection and virions were harvested using the methods outlined above for recombinant capsid production. The virions were purified by Optiprep gradient centrifugation, as above, and fractions were screened for the presence of encapsidated DNA using Quant-iT Picogreen dsDNA Reagent (Invitrogen) and by western blot for MCV VP1. The characteristics of a representative stock of native virions are shown in Figure S10.

### Native MCV infection of 293-4T cells and quantitative PCR analysis of replication

293-4T cells were detached with trypsin and 2×10^5^ cells/well were added to an untreated 96 well plate. Cells were washed once with digestion buffer (see above section on enzymatic removal of cell-surface GAGs), and then incubated for 45 minutes at 37°C with or without 5 µl each of heparinase and chondroitinase stock solution in 150 µl digestion buffer/well. Next, native MCV virions (production described above) or BKV virions (kindly provided by Gene Major, NINDS, NIH [66]) diluted in 50 µl OptiMEM were added and the cell suspensions were incubated at 37°C for an additional 45 minutes. Cells were then washed once with culture medium and again with either PBS or culture medium. The PBS suspension was collected and frozen immediately, with the goal of establishing the initial baseline number of bound MCV genomes derived from the virus inoculum. The culture medium suspension was plated in a 24 well plate and cultured for 5 to 6 days. The cultured population was trypsinized and harvested for modified Hirt extraction ([67] protocol at our laboratory website) to isolate low molecular weight DNA. Baseline samples were also subjected to modified Hirt extract. One-fiftieth of the eluted DNA sample was used in a twenty microliter reaction with DyNAmo HS SYBR Green Kit (New England Biolabs) according to manufacturer's instructions in a 7900HT Fast RT PCR System (Applied Biosystems) with ROX reference dye. The primers targeting the MCV genome are 5′-GCTTGTTAAAGGAGGAGTGG-3′ and 5′-GATCTGGAGATGATCCCTTTG-3′. The BKV-specific primers are 5′-TGGTGCTCCTGGGGCTATTGC-3′ and 5′-GCCATGCCTGATTGCTGATAGAGG-3′. A dilution series of known quantities of MCV and BKV genomic DNA were analyzed simultaneously and used to form a standard curve and calculate the number of genome copies present in each sample. An average of 12 million copies of MCV DNA and 29 million copies of BKV DNA were measured from mock-treated baseline samples collected 45 minutes after inoculation of native virions. An average of 465 million copies of MCV DNA and 895 million copies of BKV DNA were measured 5 or 6 days later. Net values conclusively showing viral amplification were calculated by subtracting the baseline number of bound viral genomes observed 45 minutes after inoculation from the number of viral genomes observed after 5 or 6 days.

### Accession numbers

Annotated nucleotide maps of all plasmids used in this work are posted on our laboratory website < http://home.ccr.cancer.gov/Lco/plasmids.asp>. The plasmids and their sequences will also be made available via Addgene.org. Accession numbers for previously-reported sequences are: MCV-R17a (HM011555), BKV-A-66H (AB369093), SLC35A1 (NM_006416).

## Supporting Information

Figure S1 **Validation of Alexa Fluor 488 conjugated capsids.** (A) A hemagglutination assay of unconjugated capsids versus capsids conjugated to Alexa Fluor 488 demonstrates that the Alexa Fluor conjugation procedure does not dramatically alter the binding properties of the capsid. (B) MCV or BKV reporter vector stocks carrying a Gaussia luciferase reporter plasmid were harvested using standard procedures or subjected to Alexa Fluor 488 conjugation then purified. The VP1 content of the resulting conjugated or unconjugated stock was determined by stained SDS-PAGE analysis, and various doses of VP1 (x-axis) were applied to A549 cells in 96 well plates for three days. Supernatants were monitored for Gaussia luciferase activity (relative light units (RLU), y-axis) using a Biolux Assay Kit (NEB). The graph shows representative results from one of two experiments. The results indicate that the transducing potential of MCV reporter vectors is not dramatically affected by the Alexa Fluor 488 conjugation procedure.
(TIF)

Figure S2 **Examples of experimental outcomes.** (A) One representative flow cytometry experiment demonstrating the effects of neuraminidase treatment of A549 cells on MCV versus BKV binding. (B) One representative experiment demonstrating the effect of neuraminidase treatment of A549 cells on MCV versus BKV reporter vector-mediated transduction of a GFP reporter gene. Blue = capsids or reporter vector on mock treated cells, green = capsids or reporter vector on neuraminidase treated cells, red = mock treated cells without virus, and orange = neuraminidase treated cells without virus.
(TIF)

Figure S3 **Effect of neuraminidase on transduction in a melanoma cell line and primary keratinocytes.** Reporter vector-mediated delivery of a GFP reporter gene in SK-MEL-2 cells or HEKa cells treated with neuraminidase was measured by flow cytometry. Results were standardized to mock-treated cells. The average of two (SK-MEL-2) or three (HEKa) separate experiments is shown and error bars represent the standard deviation.
(TIF)

Figure S4 **Transduction of Lec2 or Lec2-mslc cells pre-loaded with GT1b.** Lec2 and Lec2-mslc cells were incubated overnight with various concentrations of the ganglioside GT1b diluted in culture medium. Cells were then washed and a single dose of GFP reporter vector for the virus type indicated was added for three days. One representative experiment of three is shown.
(TIF)

Figure S5 **Inhibition of binding to A549 cells by soluble GAGs.** A549 cells were treated with roughly 50 ng Alexa Fluor 488-labled capsids pre-mixed with 0, 0.16, 4, or 100 µg/ml of heparin or chondroitin A/C in 100 µl total volume. The average relative percent mean fluorescence from three separate experiments is shown. Error bars represent the standard deviation.
(TIF)

Figure S6 **Sulfation is required for MCV transduction in a melanoma cell line.** SK-MEL-2 cells were adapted to growth in 50 mM sodium chlorate. MCV and BKV transduction in SK-MEL-2 cells grown in medium with our without chlorate was compared side-by-side using the same dose of reporter vector. The average percent of GFP positive cells after three days from three separate experiments is shown and error bars represent the standard error of the mean.
(TIF)

Figure S7 **Verification of enzyme activity and specificity.** A549 cells were resuspended with PBS supplemented with10 mM EDTA, washed and treated with chondroitinase ABC (“CSase”) or with heparinase I/III (“HSase”), or with both. Monoclonal antibodies to HS (10E4) or CS (CS-56) were then incubated with the treated cells. The cells were then washed, incubated with a fluorescently-conjugated secondary antibody, then subjected to flow cytometric analysis. The mean fluorescence relative to “Mock” treatment was determined and the average of two separate experiments is shown. Error bars represent standard deviation.
(TIF)

Figure S8 **MCV entry requires cell surface glycosaminoglycans on melanoma cells and keratinocytes.** SK-MEL-2 cells or HEKa cells were treated with chondroitinase ABC (“CSase”) or with heparinase I/III (“HSase”), or with both HSase and CSase prior to inoculation with reporter vectors. The average of three (SK-MEL-2) or four (HEKa) separate experiments is shown and error bars represent the standard deviation.
(TIF)

Figure S9 **Neuraminidase treatment of pgsA-745 cells.** The binding of Alexa Fluor 488-conjugated capsids (A) or reporter vector-mediated delivery of a GFP reporter gene (B) to GAG-deficient pgsA-745 cells treated with neuraminidase was measured by flow cytometry. MCV binding and transduction were performed in the presence of 20 µg/ml heparin. Results were standardized to mock treatment. The average of three separate experiments is shown and error bars represent the standard deviation.
(TIF)

Figure S10 **Propagation of native MCV.** A subconfluent 75 cm^2^ flask of 293-4T cells was infected with 50 µl (approximately 50 billion genome copies) of purified MCV virions produced by transfection of 293-TT cells with recombinant MCV isolate R17a genomic DNA. The infected cells were expanded into in two 225 cm^2^ flasks. At each time point shown, 4/5ths of the culture was harvested and virions were extracted from the cells and subjected to Optiprep gradient purification. The remaining 1/5^th^ of the culture was subjected to ongoing propagation. Fractions of Optiprep gradients were collected and screened by Western blot for the presence of VP1 and by qPCR for the presence of MCV genomic DNA. The peak fractions were collected and pooled. A 10 µl aliquot of each pool of peak fractions was examined by Western blot for VP1 alongside a 10 µl aliquot of the native virus used to infect the 293-4T cells (“Input MCV”). Known quantities of recombinant MCV capsids were also compared in this Western blot. Samples of each harvest were also digested with proteinase K and the DNA was purified for analysis of genome copy number by qPCR.
(TIF)
