# Supplementary figures and images for: Glycosaminoglycans and Sialylated Glycans Sequentially Facilitate Merkel Cell Polyomavirus Infectious Entry
Source: PLoS Pathog. 2011 Jul 28;7(7):e1002161. doi: 10.1371/journal.ppat.1002161 (PMC3145800; doi:10.1371/journal.ppat.1002161)

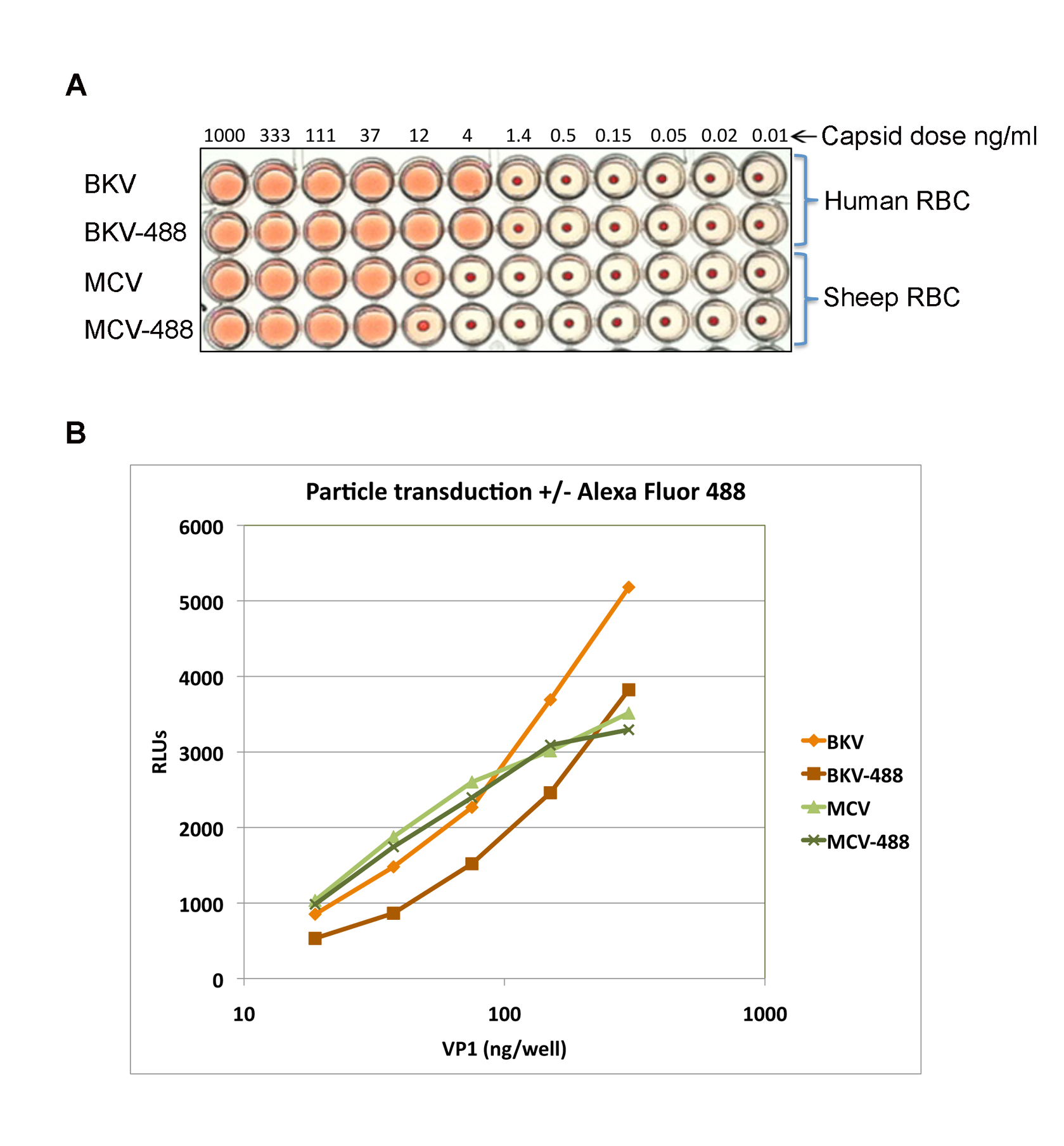

Supplement: Figure S1 — Validation of Alexa Fluor 488 conjugated capsids. (A) A hemagglutination assay of unconjugated capsids versus capsids conjugated to Alexa Fluor 488 demonstrates that the Alexa Fluor conjugation procedure does not dramatically alter the binding properties of the capsid. (B) MCV or BKV reporter vector stocks carrying a Gaussia luciferase reporter plasmid were harvested using standard procedures or subjected to Alexa Fluor 488 conjugation then purified. The VP1 content of the resulting conjugated or unconjugated stock was determined by stained SDS-PAGE analysis, and various doses of VP1 (x-axis) were applied to A549 cells in 96 well plates for three days. Supernatants were monitored for Gaussia luciferase activity (relative light units (RLU), y-axis) using a Biolux Assay Kit (NEB). The graph shows representative results from one of two experiments. The results indicate that the transducing potential of MCV reporter vectors is not dramatically affected by the Alexa Fluor 488 conjugation procedure. (TIF) [file ppat.1002161.s001.tif]

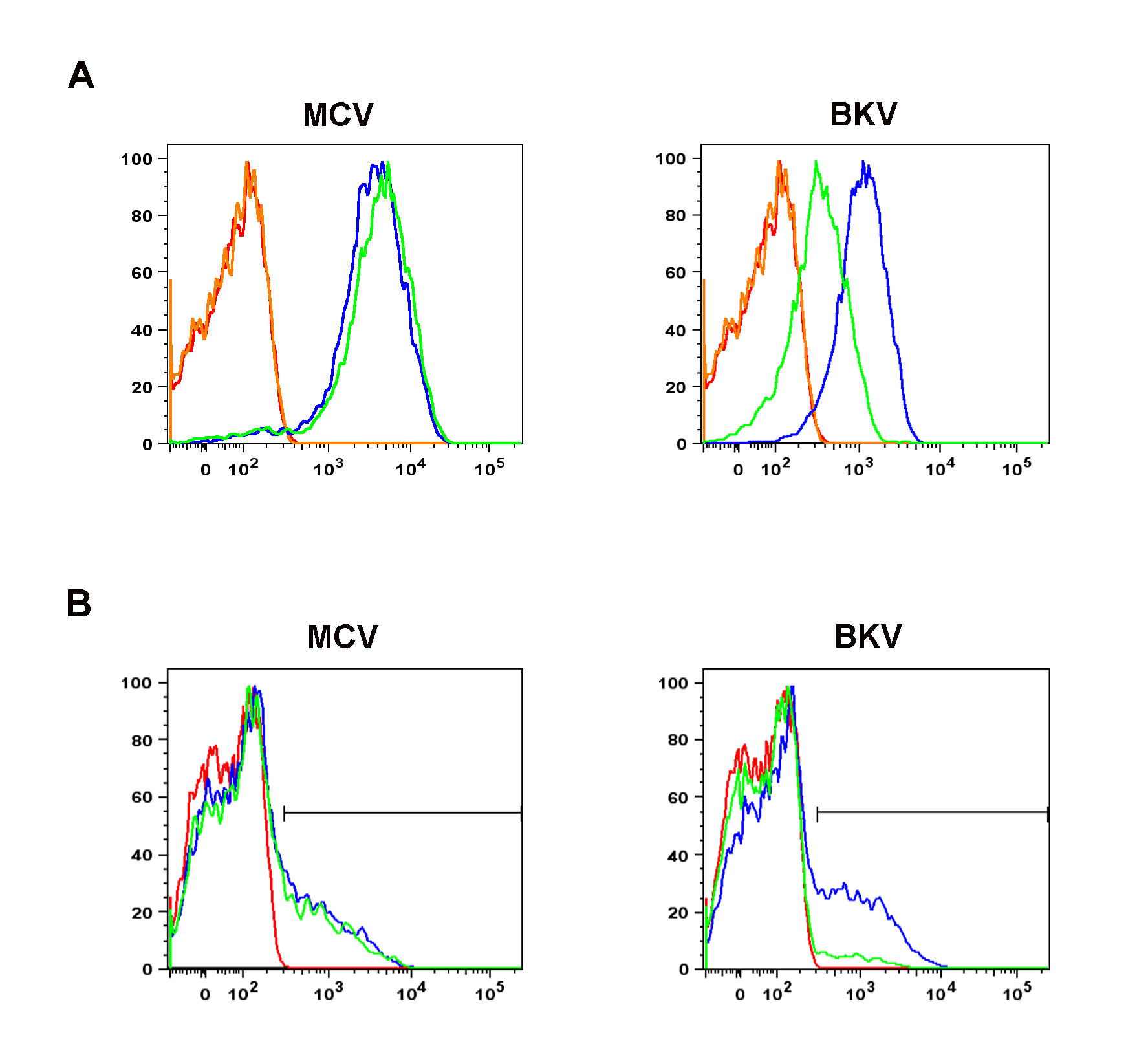

Supplement: Figure S2 — Examples of experimental outcomes. (A) One representative flow cytometry experiment demonstrating the effects of neuraminidase treatment of A549 cells on MCV versus BKV binding. (B) One representative experiment demonstrating the effect of neuraminidase treatment of A549 cells on MCV versus BKV reporter vector-mediated transduction of a GFP reporter gene. Blue = capsids or reporter vector on mock treated cells, green = capsids or reporter vector on neuraminidase treated cells, red = mock treated cells without virus, and orange = neuraminidase treated cells without virus. (TIF) [file ppat.1002161.s002.tif]

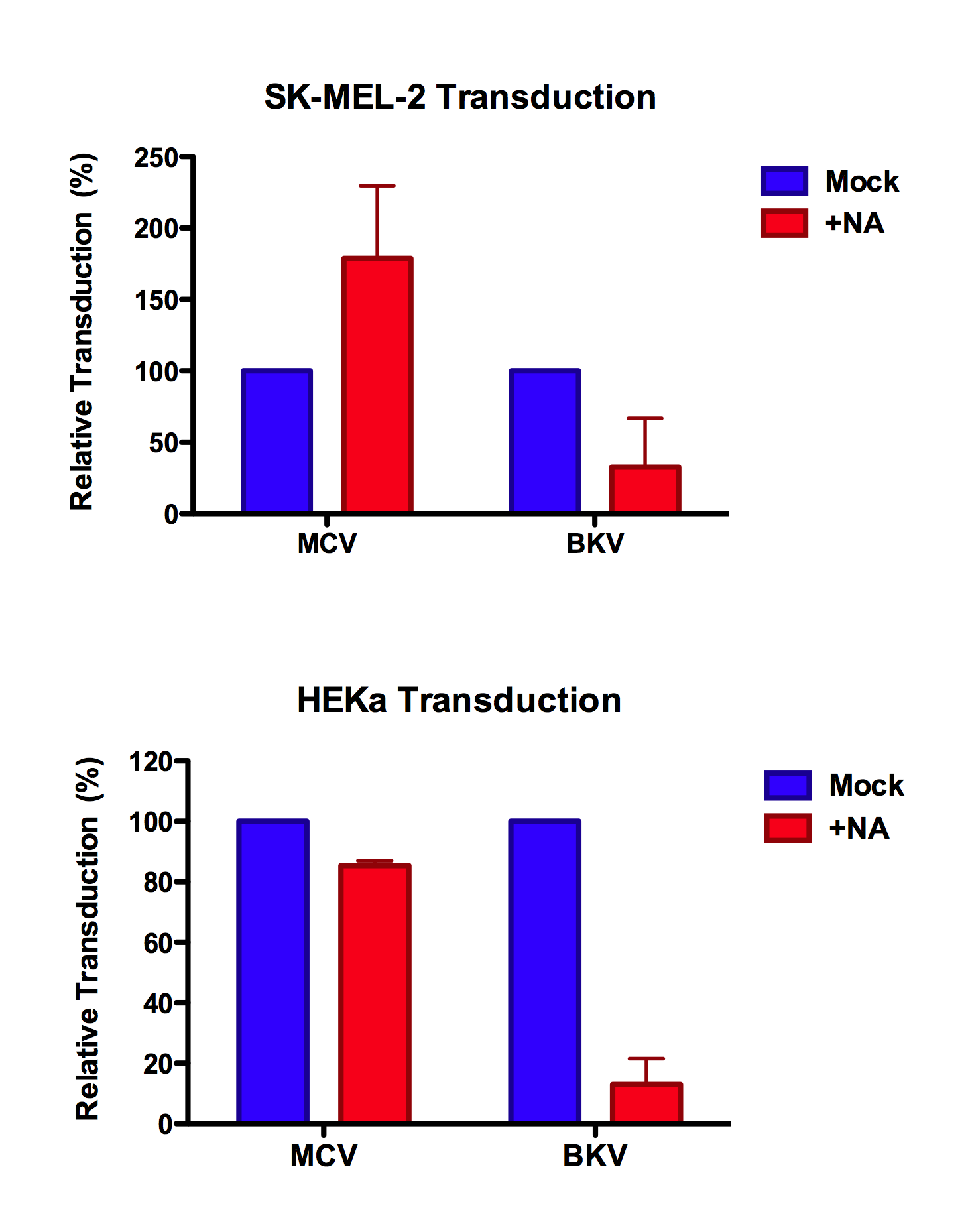

Supplement: Figure S3 — Effect of neuraminidase on transduction in a melanoma cell line and primary keratinocytes. Reporter vector-mediated delivery of a GFP reporter gene in SK-MEL-2 cells or HEKa cells treated with neuraminidase was measured by flow cytometry. Results were standardized to mock-treated cells. The average of two (SK-MEL-2) or three (HEKa) separate experiments is shown and error bars represent the standard deviation. (TIF) [file ppat.1002161.s003.tif]

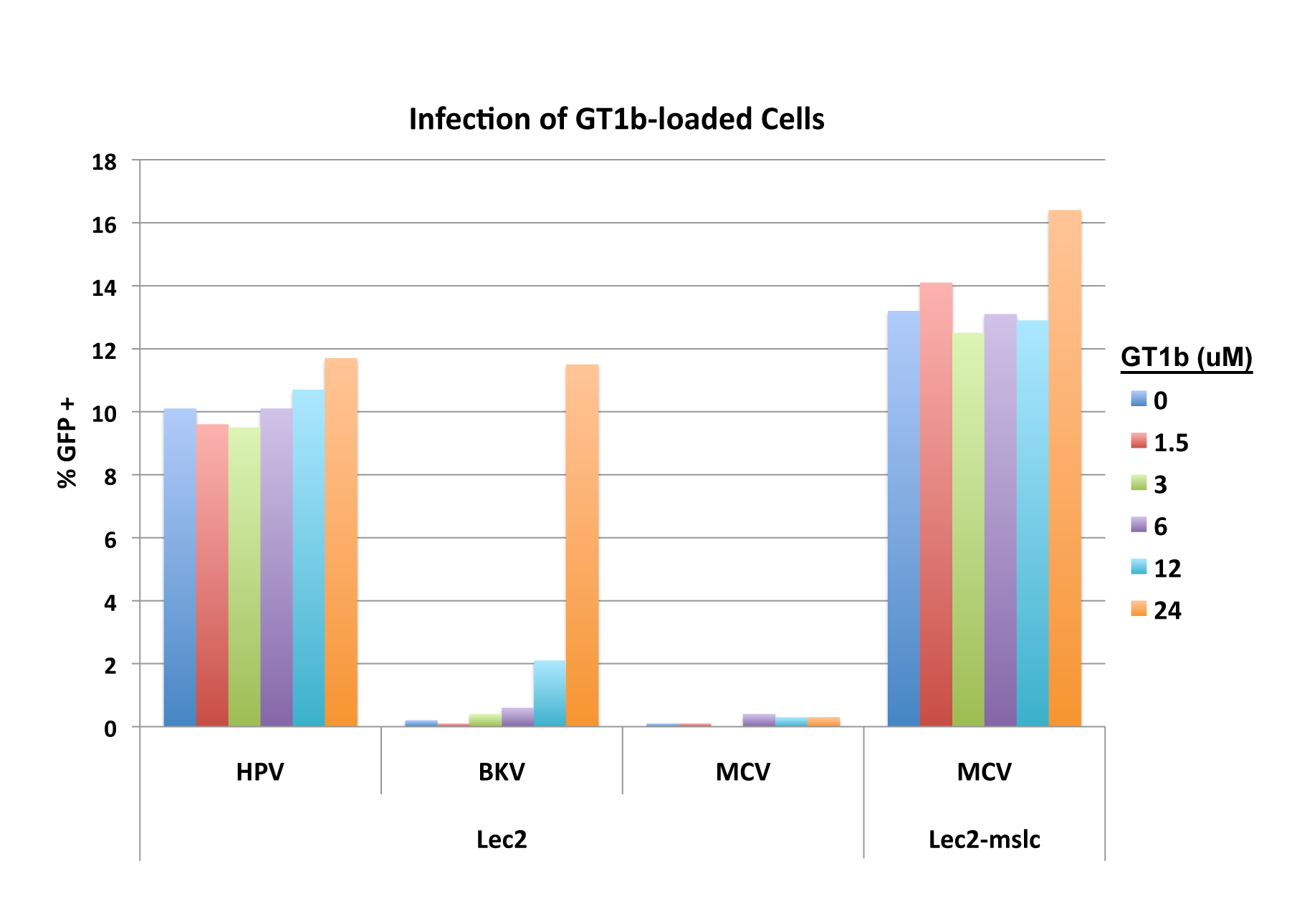

Supplement: Figure S4 — Transduction of Lec2 or Lec2-mslc cells pre-loaded with GT1b. Lec2 and Lec2-mslc cells were incubated overnight with various concentrations of the ganglioside GT1b diluted in culture medium. Cells were then washed and a single dose of GFP reporter vector for the virus type indicated was added for three days. One representative experiment of three is shown. (TIF) [file ppat.1002161.s004.tif]

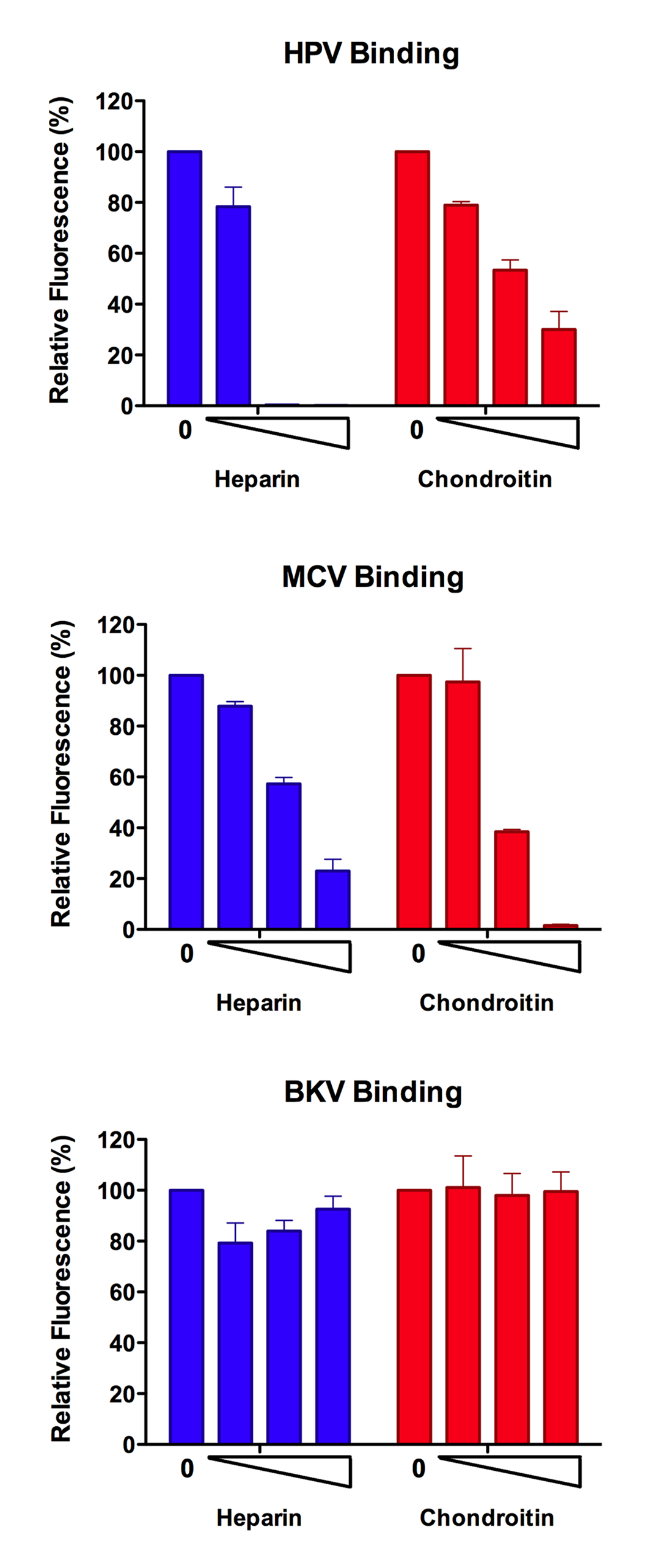

Supplement: Figure S5 — Inhibition of binding to A549 cells by soluble GAGs. A549 cells were treated with roughly 50 ng Alexa Fluor 488-labled capsids pre-mixed with 0, 0.16, 4, or 100 µg/ml of heparin or chondroitin A/C in 100 µl total volume. The average relative percent mean fluorescence from three separate experiments is shown. Error bars represent the standard deviation. (TIF) [file ppat.1002161.s005.tif]

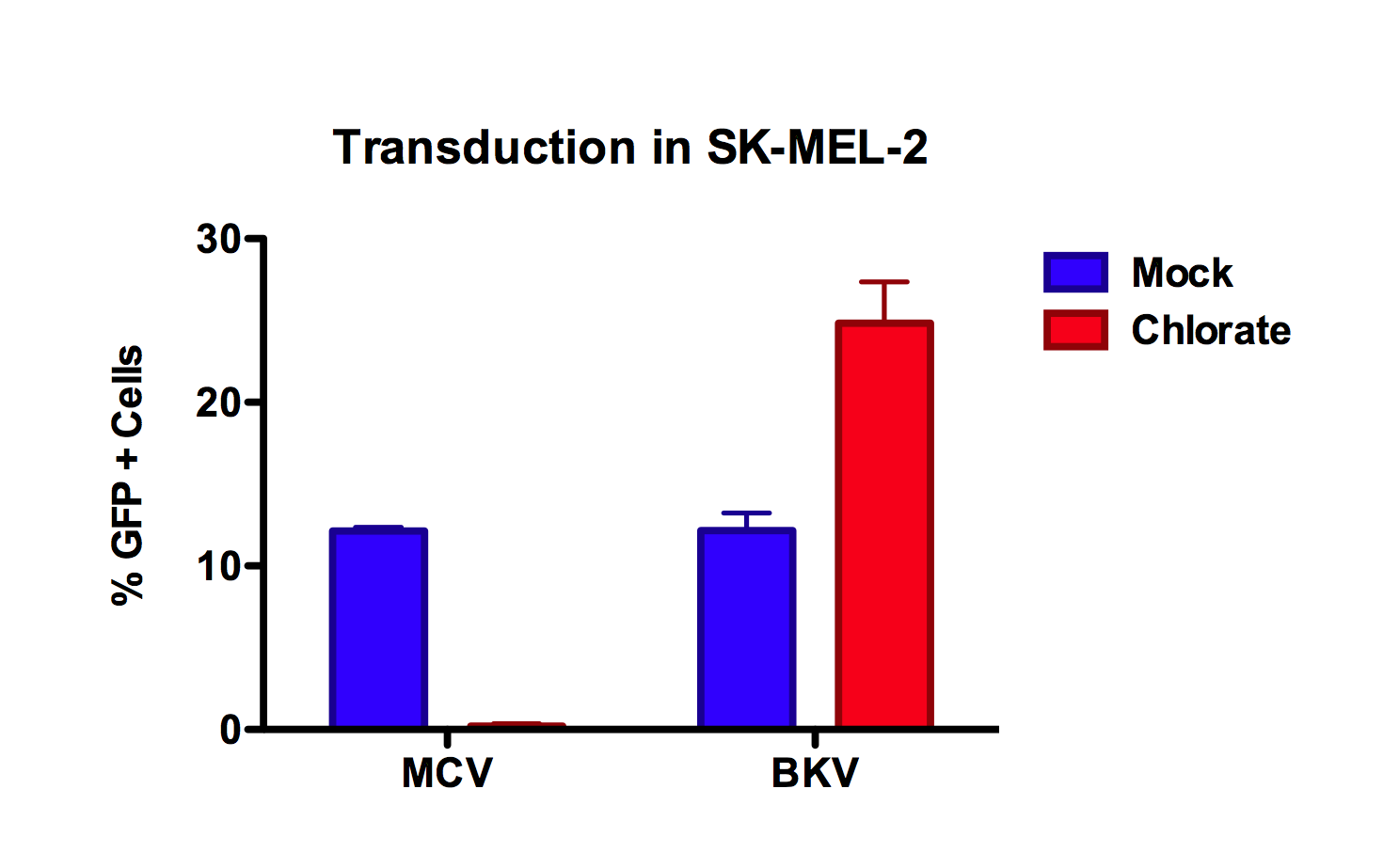

Supplement: Figure S6 — Sulfation is required for MCV transduction in a melanoma cell line. SK-MEL-2 cells were adapted to growth in 50 mM sodium chlorate. MCV and BKV transduction in SK-MEL-2 cells grown in medium with our without chlorate was compared side-by-side using the same dose of reporter vector. The average percent of GFP positive cells after three days from three separate experiments is shown and error bars represent the standard error of the mean. (TIF) [file ppat.1002161.s006.tif]

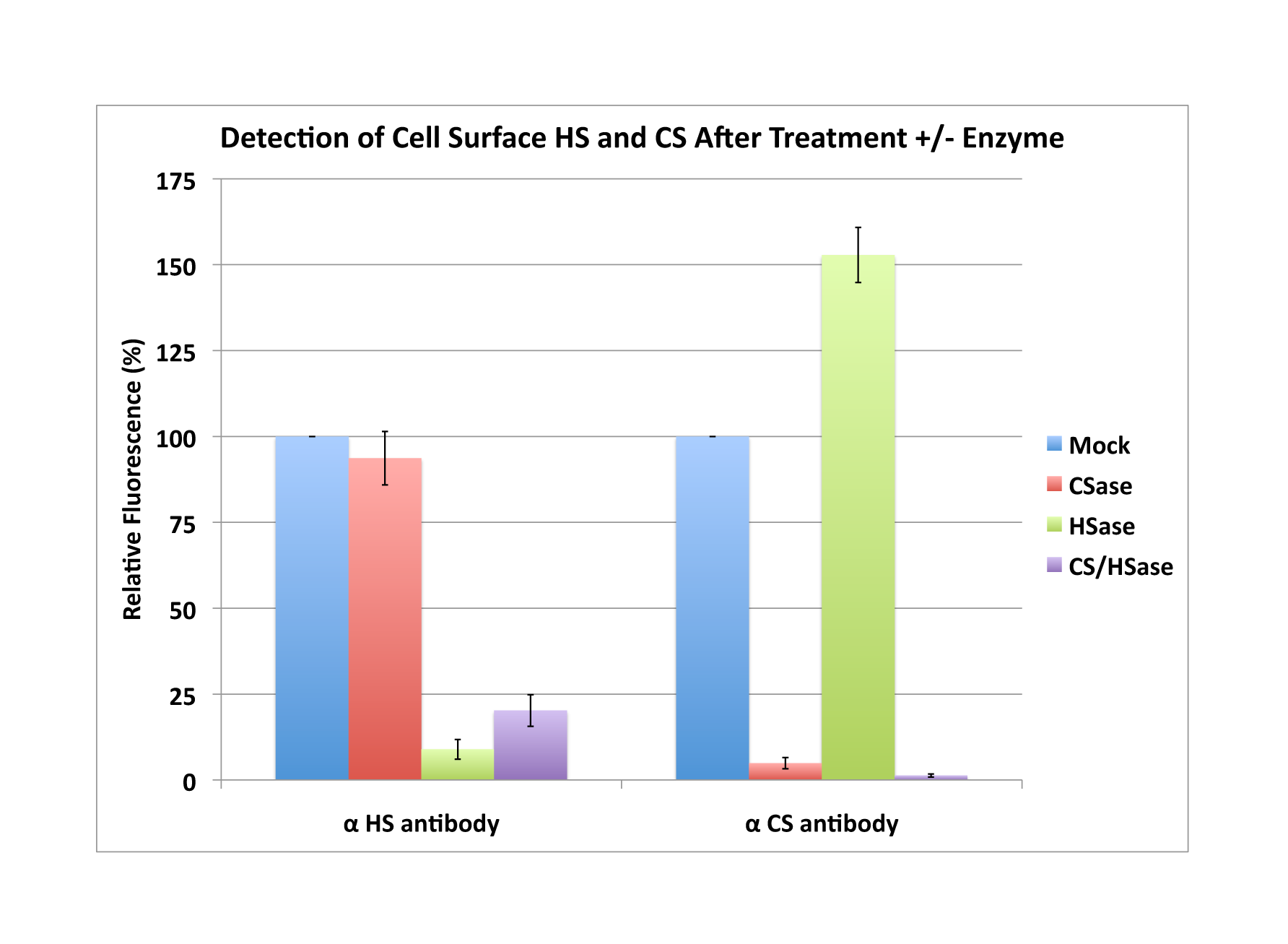

Supplement: Figure S7 — Verification of enzyme activity and specificity. A549 cells were resuspended with PBS supplemented with10 mM EDTA, washed and treated with chondroitinase ABC (“CSase”) or with heparinase I/III (“HSase”), or with both. Monoclonal antibodies to HS (10E4) or CS (CS-56) were then incubated with the treated cells. The cells were then washed, incubated with a fluorescently-conjugated secondary antibody, then subjected to flow cytometric analysis. The mean fluorescence relative to “Mock” treatment was determined and the average of two separate experiments is shown. Error bars represent standard deviation. (TIF) [file ppat.1002161.s007.tif]

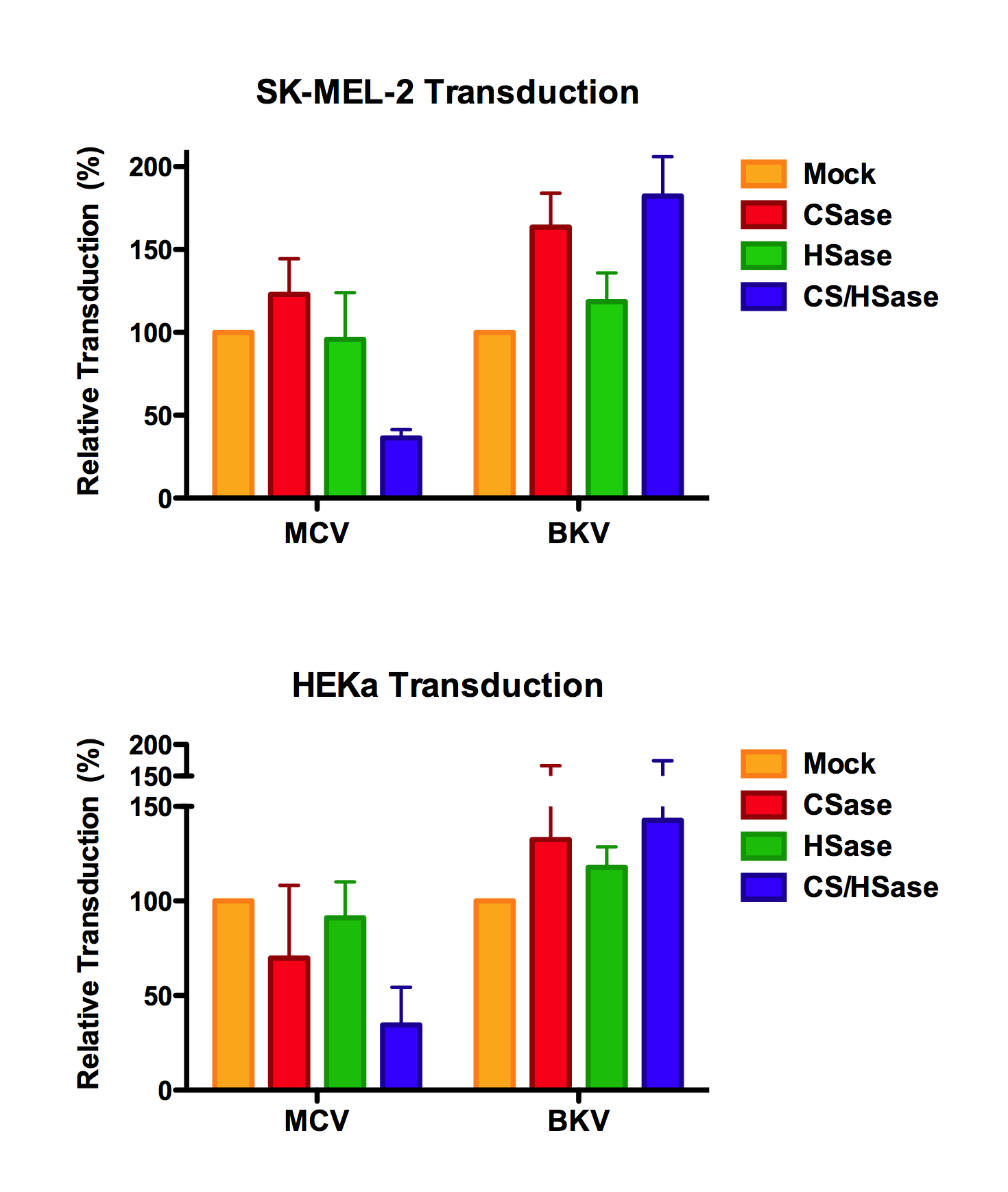

Supplement: Figure S8 — MCV entry requires cell surface glycosaminoglycans on melanoma cells and keratinocytes. SK-MEL-2 cells or HEKa cells were treated with chondroitinase ABC (“CSase”) or with heparinase I/III (“HSase”), or with both HSase and CSase prior to inoculation with reporter vectors. The average of three (SK-MEL-2) or four (HEKa) separate experiments is shown and error bars represent the standard deviation. (TIF) [file ppat.1002161.s008.tif]

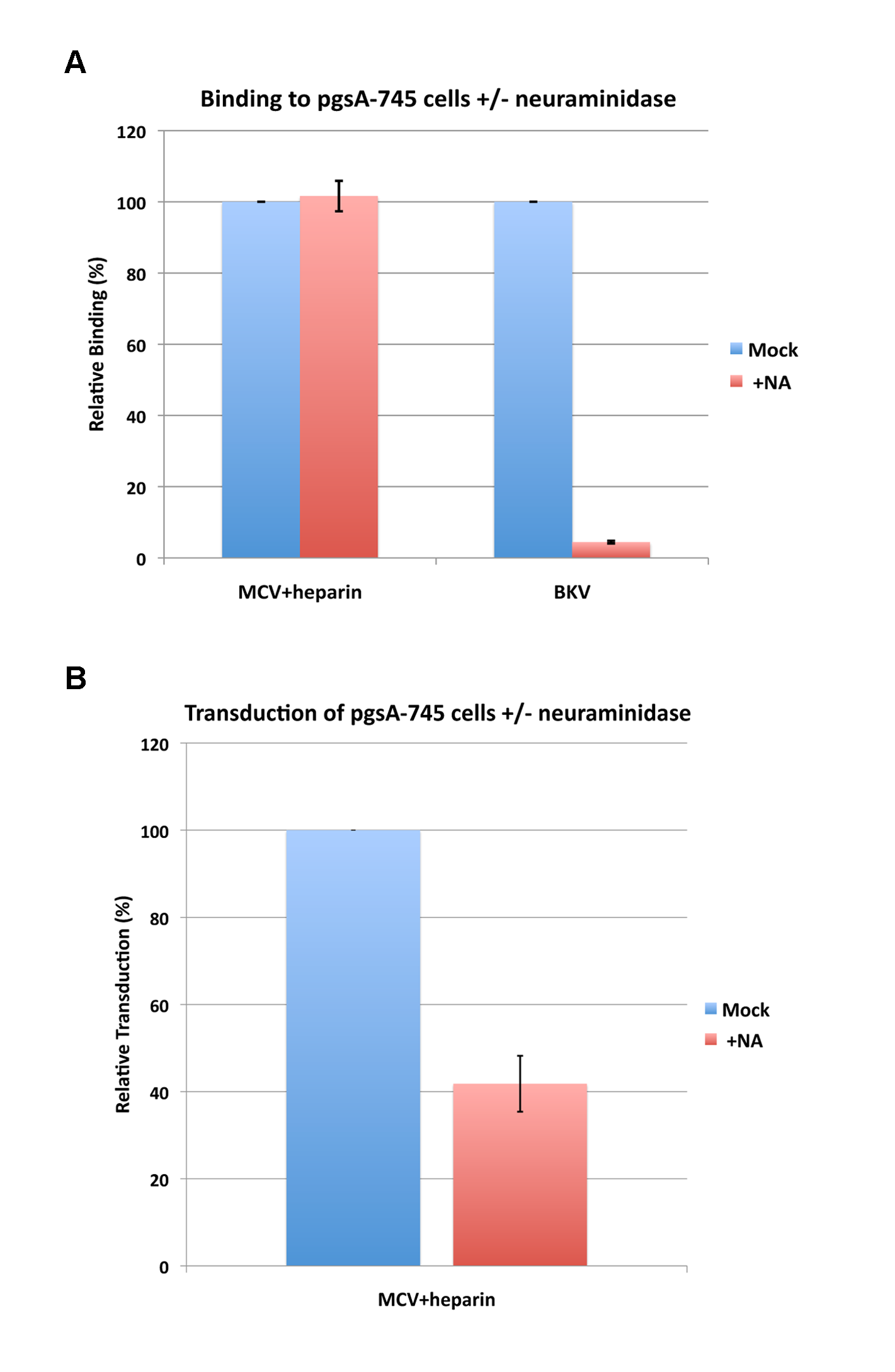

Supplement: Figure S9 — Neuraminidase treatment of pgsA-745 cells. The binding of Alexa Fluor 488-conjugated capsids (A) or reporter vector-mediated delivery of a GFP reporter gene (B) to GAG-deficient pgsA-745 cells treated with neuraminidase was measured by flow cytometry. MCV binding and transduction were performed in the presence of 20 µg/ml heparin. Results were standardized to mock treatment. The average of three separate experiments is shown and error bars represent the standard deviation. (TIF) [file ppat.1002161.s009.tif]

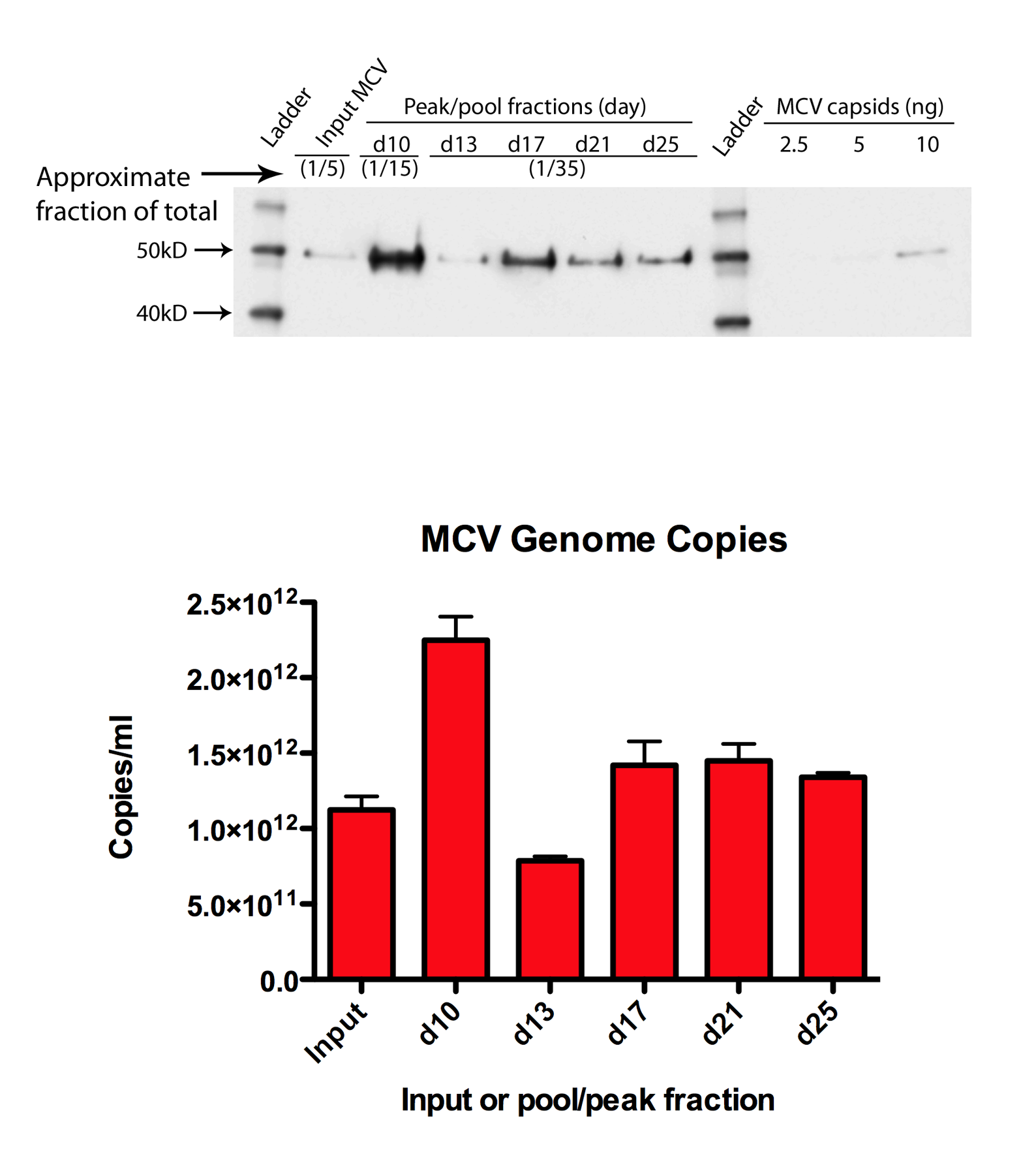

Supplement: Figure S10 — Propagation of native MCV. A subconfluent 75 cm2 flask of 293-4T cells was infected with 50 µl (approximately 50 billion genome copies) of purified MCV virions produced by transfection of 293-TT cells with recombinant MCV isolate R17a genomic DNA. The infected cells were expanded into in two 225 cm2 flasks. At each time point shown, 4/5ths of the culture was harvested and virions were extracted from the cells and subjected to Optiprep gradient purification. The remaining 1/5th of the culture was subjected to ongoing propagation. Fractions of Optiprep gradients were collected and screened by Western blot for the presence of VP1 and by qPCR for the presence of MCV genomic DNA. The peak fractions were collected and pooled. A 10 µl aliquot of each pool of peak fractions was examined by Western blot for VP1 alongside a 10 µl aliquot of the native virus used to infect the 293-4T cells (“Input MCV”). Known quantities of recombinant MCV capsids were also compared in this Western blot. Samples of each harvest were also digested with proteinase K and the DNA was purified for analysis of genome copy number by qPCR. (TIF) [file ppat.1002161.s010.tif]
